# *FaTEDT1L* of Octoploid Cultivated Strawberry Functions as a Transcriptional Activator and Enhances Abiotic Stress Tolerance in Transgenic *Arabidopsis*

**DOI:** 10.3390/ijms251810091

**Published:** 2024-09-19

**Authors:** Ching-Ying Chu, Lee-Fong Lin, Shang-Chih Lai, Jui-Hung Yang, Ming-Lun Chou

**Affiliations:** 1 Department of Life Sciences, Tzu Chi University, Hualien 97004, Taiwan; 108726104@gms.tcu.edu.tw (C.-Y.C.); leelin@gms.tcu.edu.tw (L.-F.L.); 2 Department of Biomedical Sciences and Engineering, Tzu Chi University, Hualien 97004, Taiwan; 3 School of Post-Baccalaureate Chinese Medicine, Tzu Chi University, Hualien 97004, Taiwan; edward6174@mail.tcu.edu.tw; 4 Biomedical Technology and Device Research Laboratories, Industrial Technology Research Institute, Hsinchu 30011, Taiwan; jhyen@itri.org.tw

**Keywords:** FaTEDT1L, HD-ZIP IV, osmotic stress, octoploid cultivated strawberry, transgenic *Arabidopsis*

## Abstract

Plants may encounter abiotic stresses, such as drought, flooding, salinity, and extreme temperatures, thereby negatively affecting their growth, development, and reproduction. In order to enhance their tolerance to such stresses, plants have developed intricate signaling networks that regulate stress-responsive gene expression. For example, *Arabidopsis Enhanced Drought Tolerance1/HOMEODOMAIN GLABROUS 11* (*AtEDT1/HDG11*), one of the transcription factor genes from the group IV of homeodomain-leucine zipper (HD-ZIP) gene family, has been shown to increase drought tolerance in various transgenic plants. However, the underlying molecular mechanisms of enhanced stress tolerance remain unclear. In this study, we identified a homologous gene related to *AtEDT1/HDG11*, named *FaTEDT1L*, from the transcriptome sequencing database of cultivated strawberry. Phylogenetic analysis revealed the close relationship of FaTEDT1L with AtEDT1/HDG11, which is one of the group IV members of the HD-ZIP gene family. Yeast one-hybrid analysis showed that FaTEDT1L functions as a transcriptional activator. Transgenic *Arabidopsis* plants overexpressing *FaTEDT1L* under the control of the cauliflower mosaic virus (CaMV) 35S promoter exhibited significantly enhanced tolerance to osmotic stress (both drought and salinity) when compared to the wild-type (WT) plants. Under osmotic stress, the average root length was 3.63 ± 0.83 cm, 4.20 ± 1.03 cm, and 4.60 ± 1.14 cm for WT, *35S::FaTEDT1L T_2_ #3*, and *35S:: FaTEDT1L T_2_ #5*, respectively. Substantially increased root length in *35S::FaTEDT1L T_2_ #3* and *35S::FaTEDT1L T_2_ #5* was noted when compared to the WT. In addition, the average water loss rates were 64%, 57.1%, and 55.6% for WT, *35S::FaTEDT1L T2 #3*, and *35S::FaTEDT1L T_2_ #5*, respectively, after drought treatment, indicating a significant decrease in water loss rate of *35S:: FaTEDT1L T_2_ #3* and *35S::FaTEDT1L T_2_ #5* is a critical factor in enhancing plant drought resistance. These findings thus highlight the crucial role of *FaTEDT1L* in mitigating drought and salt stresses and regulating plant osmotic stress tolerance. Altogether, *FaTEDT1L* shows its potential usage as a candidate gene for strawberry breeding in improving crop resilience and increasing agricultural productivity under adverse environmental conditions.

## 1. Introduction

In the realm of plant biology, stress can be broadly categorized into biotic and abiotic stresses [1,2]. Abiotic stress is associated with environmental factors such as temperature fluctuations (heat, cold, and frost damage), water-related challenges (drought, salinity, and waterlogging), as well as pollutants like pesticides and industrial toxins, leading to tissue browning, decay, and even plant death [3]. With global warming and climate change exacerbating these challenges, the resilience required by plants to endure such stress is expected to escalate [4].

When plants perceive stress, stress factors may serve as triggers to initiate intracellular signaling pathways. Activation of specific transcription factors by these stress factors leads to their binding to the promoter regions of downstream target genes [5,6]. Through collaborative interactions between these transcription factors and target genes, the expression of the target genes is activated, thereby equipping plants with the ability to withstand stress [7,8]. Previous studies have identified some key transcription factors involved in the above-mentioned molecular pathways [9,10]. Researchers have subsequently introduced and overexpressed these transcription factors in experimental materials in order to investigate their potential role in enhanced stress tolerance of plants [11,12]. Thus, this experimental strategy aims to gain more molecular insights regarding plant resilience to stress by examining the effects of these transcription factors under adverse conditions.

The HD-Zip family comprises plant-specific transcription factors [13,14,15,16,17,18,19,20]. These factors possess a conserved 60 amino acid sequence known as the homeodomain (HD), which facilitates DNA binding and is highly conserved across species [21]. Additionally, HD contains a leucine zipper domain (LZ) composed of two α-helical monomers with leucine residues [22], thereby named HD-Zip. HD-Zip transcription factors are classified into four distinct groups (HD-Zip I, HD-Zip II, HD-Zip III, and HD-Zip IV) based on protein sequence, structure, conserved regions, and functions [18,19,20]. The HD-Zip group I (17 members) primarily participates in plant responses to abiotic stresses, including light and water stress [23,24]. Some members regulate hypocotyl elongation, branch formation, or flowering time, and others may act as regulators in abscisic acid (ABA) and sucrose signaling [17,25,26,27]. The HD-Zip group II (10 members) is involved in phototropic and auxin responses, impacting the morphology of plants, germination, and leaf photosynthesis [18,19,28,29]. The HD-Zip group III (5 members) plays a pivotal role in diverse developmental processes, including vascular tissue differentiation, lateral organ polarity determination, embryonic morphology, and meristematic tissue initiation [30,31,32,33,34]. Lastly, the HD-Zip group IV (16 members) mainly regulates trichome formation and morphological development, with a few members influencing floral organ characteristics and seed germination [17,35,36,37,38].

Recent advancements in Next Generation Sequencing (NGS) technology have facilitated the efficient identification of novel genes in species through high-throughput sequencing. RNA-seq analysis, particularly de novo assembly, has become a valuable approach for studying non-model organisms and their transcriptomes [39,40]. Even in the absence of a reference genome, this method allows for the identification of unknown novel genes [41]. The diploid cultivated strawberry species *Fragaria vesca* has both genomic and transcriptomic resources available online [42,43]. In addition, our previous research has established a transcriptome sequencing database for the octoploid cultivated strawberry *F*. × *ananassa* Duchessne cv. *Toyonoka* and the heat shock transcription factor (HSF) gene family were successfully identified, demonstrating their association with plant heat tolerance [44].

In this study, we utilized the above-mentioned transcriptome sequencing database of the octoploid cultivated strawberry, and a homologous gene, *FaTEDT1L*, which shares very high similarity with *AtEDT1*/*HDG11*, was identified. Subsequently, we aimed to explore the relationship between *FaTEDT1L* and stress tolerance. Through sequence alignment and phylogenetic analysis, we classified FaTEDT1L as an HD-ZIP group IV protein. The presence of an activation domain within FaTEDT1L was discovered and this domain functions similarly to a transcription factor, i.e., it can activate downstream gene expression. Additionally, we generated transgenic *Arabidopsis* plants that overexpressed *FaTEDT1L*. These transgenic plants were found to exhibit increased resistance to osmotic stress, characterized by enhanced root development and reduced water loss. At the molecular level, we also provided evidence that the overexpression of *FaTEDT1L* upregulated the expression of osmotic stress-related genes, such as *SOS* (*SOS1*, *SOS2*, and *SOS3*), thereby enhancing the transgenic plants’ tolerance to drought and salt stresses.

## 2. Results and Discussion

### 2.1. Isolation and Characterization of FaTEDT1L

The *Arabidopsis thaliana* mutant *edt1* (*enhanced drought tolerance 1*) has provided insights into the role of the overexpressed gene in improving plant tolerance to drought stress. It was subsequently identified as *HDG11*, a member of the HD-ZIP group IV. Recent research has focused on ectopically expressing *AtEDT1*/*HDG11* in various plant species in order to investigate its impact on stress tolerance [45]. Promising results have been obtained, including increased drought and salt tolerance in tall fescue (*Festuca arundinacea*) [46], improved drought resistance in rice without compromising grain yield [47], enhanced salt and drought tolerance as well as increased cotton yield in upland cotton (*Gossypium hirsutum*) and poplar (*Populus tomentosa*) [48], elevated drought tolerance and resistance to osmotic stress in *Brassica oleracea* var. *alboglabra* [49], augmented drought tolerance in *Medicago sativa* [50], and significantly enhanced salt and drought tolerance in peanut (*Arachis hypogaea*) [51]. Altogether, these data therefore support the potential role of AtEDT1/HDG11 in enhancing resistance to abiotic stress, particularly drought stress, across diverse plant species [52,53].

Following activation of the *AtEDT1*/*HDG11* gene by using the cauliflower mosaic virus 35S enhancer, transgenic plants have successfully shown enhanced tolerance to drought [39]. We thus hypothesized that transgenic plants would also exhibit a similar effect of increased drought and salt tolerance if we isolate an *EDT1* homologous gene and ectopically express this gene abundantly in *A. thaliana*. To test this hypothesis, we first input the full-length sequence of the *AtEDT1*/*HDG11* gene into the octoploid cultivated strawberry by using the transcriptome sequencing database [44], and a homologous gene was identified and named *F. × ananassa* Duchessne cv. *Toyonoka enhanced drought tolerance 1* (*FaTEDT1*). To confirm this assembled *FaTEDT1* could be expressed in plant tissues, total RNA from the foliar tissue of octoploid cultivated strawberry was extracted. RT-PCR was performed by using designed gene-specific primers based on the assembled *FaTEDT1* sequences (Table S1). After amplification, the full-length gene fragment with the expected size was obtained. We further compared the sequencing results of *FaTEDT1* obtained from an RT-PCR reaction [FaTEDT1 (RT-PCR)] with the *FaTEDT1* gene sequences acquired from the transcriptome database [FaTEDT1 (T)] via sequence alignment analysis. The comparison revealed significant inconsistencies at the 3′ end region between these two sequences, leading us to suspect an assembly error in the database (Figure S1). Consequently, we renamed the full-length sequence gained from the total RNA extracted from strawberry foliar tissue through RT-PCR as *FaTEDT1L* (*F. × ananassa* Duchessne cv. Toyonoka *enhanced drought tolerance 1-like* gene). The sequenced cDNA has a total length of 2130 bps and encodes a protein of 709 amino acids (GenBank accession number: PQ120428) (Figure S2).

To confirm that the different sequences at the 3′ end between the assembled FaTEDT1 (T) and FaTEDT1L RT-PCR transcripts were likely due to the assembled errors during database construction, we inputted the *FaTEDT1L* gene sequences into the Phytozome v13 website [https://phytozome-next.jgi.doe.gov/ (accessed on 24 March 2024)] to blast the assembled and annotated genome dataset. As a result, a homologous gene (FvH4_5g18090) from the diploid strawberry (*F. vesca*) was obtained and named *FvEDT1*. Subsequently, we performed amino acid sequence alignment between the FaTEDT1L of octoploid strawberry and the FvEDT1 of diploid strawberry. Our results revealed a 99.4% similarity between these two gene products, indicating FaTEDT1L and FvEDT1 share very high homology at both N-terminal and C-terminal ends (Figure S3). However, although our data suggest a close relationship between the *FaTEDT1* (RT-PCR and T) from the octoploid and the *FvEDT1* from the diploid strawberries in terms of gene homology, a significant difference between *FaTEDT1* (T) and *FvEDT1* near the 3′ end was found (Figures S1 and S3). Taken together, we currently cannot rule out the possibility that the assembled *FaTEDT1* (T) gene through transcriptome sequencing has a lower expression level in the extracted leaf materials, leading to undetectable mRNA and/or unamplified PCR products by RT-PCR reactions. Further investigations are necessary in order to validate and clarify the expression patterns of *FaTEDT1* (T) in octoploid strawberries. Therefore, the resulting *FaTEDT1L* RT-PCR transcripts were chosen for the subsequent analysis in this study.

### 2.2. Classification of FaTEDT1L as a Member of the HD-ZIP Group IV Gene Family

**Figure 1:**
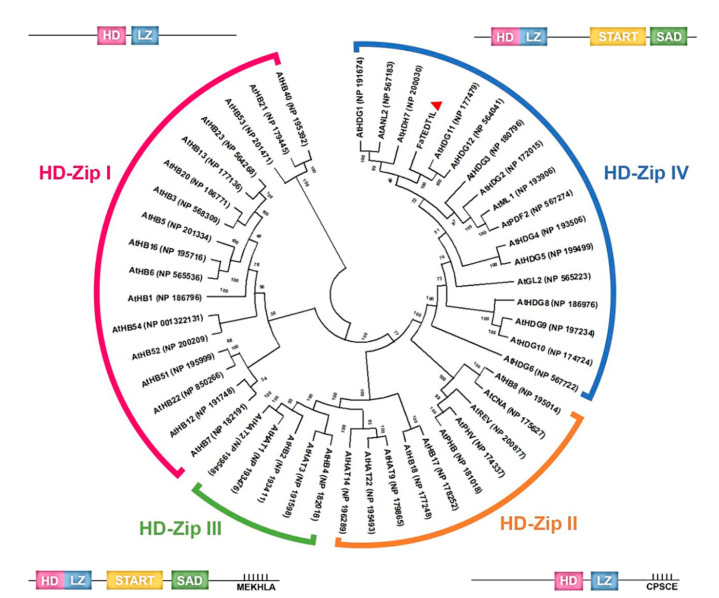
Evolutionary phylogenetic tree depicting the relationship between FaTEDT1L and the currently identified HD-Zip gene families present in *Arabidopsis*. Four different color backgrounds indicate the four distinct groups within the *Arabidopsis* HD-Zip gene families based on gene sequences and structure. The red “▲” symbol shows the position of FaTEDT1L, which is classified to be an HD-Zip IV group member according to the branching pattern of the evolutionary phylogenetic tree.

There are 48 genes so far in the *Arabidopsis* genome that have been identified to encode the HD-ZIP domain. These gene products are classified into four distinct groups, namely HD-Zip I, HD-Zip II, HD-Zip III, and HD-Zip IV, based on their protein sequences, structures, highly conserved regions, and biological functions [18,19,20]. Among these groups, the HD-Zip IV family comprises 16 genes, including *GLABRA2* (*GL2*), *ANTHOCYANINLESS2* (*ANL2*), *FLOWERING LATE* (*FWA*), *ARABIDOPSIS THALIANA MERISTEM LAYER1 (ATML1)*, *PROTODERMAL FACTOR2 (PDF2)*, *HDG1* to *HDG5*, and *HDG7* to *HDG12*. To analyze their evolutionary relationship, we performed a neighbor-joining (NJ) algorithm with 1000 bootstrap replicates by using Molecular Evolutionary Genetic Analysis (MEGA) X software version 1.54k 15 September 2024 [54]. In this analysis, we included the protein sequences of the 48 HD-ZIP members from *Arabidopsis*, along with the protein sequence of FaTEDT1L. Thus, a phylogenetic tree was constructed. The results of this analysis revealed that the *Arabidopsis* HD-Zip gene clusters can be divided into four distinct groups: HD-Zip I (17 members), HD-Zip II (10 members), HD-Zip III (5 members), and HD-Zip IV (16 members) (Figure 1). In addition, FaTEDT1L was classified as an HD-Zip group IV member and showed a close evolutionary relationship with *Arabidopsis* AtHDG11 and AtHDG12 based on the phylogenetic analysis (Figure 1).

The HD-Zip I and HD-Zip II families share very similar structures, both containing highly conserved HD and LZ functional domains at the N-terminus. Of note, the HD-Zip II family possesses a unique CPSCE (Cys, Pro, Ser, Cys, Glu) functional domain at the C-terminus, while HD-Zip I does not. The CPSCE domain facilitates the formation of multimeric proteins and is responsible for intermolecular bonding between cysteine residues (Cys-Cys) [55]. Despite their structural similarities, the HD-Zip I and HD-Zip II families differ in their specific target sites on downstream genes, primarily due to amino acid variations that affect their interactions with DNA molecules. The HD-Zip III and HD-Zip IV families are characterized by highly conserved START (steroidogenic acute regulatory protein-related lipid transfer) and SAD (START-associated domain) regions following the HD-Zip domain. The START region, composed of about 200 amino acid residues, is known to be involved in lipid/steroid transport in animals, although its function in plants remains unclear. Some researchers speculate that it may regulate functions related to lipid/steroid-associated proteins in plants [55]. The HD-Zip III family is further distinguished by the presence of a MEKHLA (Met, Glu, Lys, His, Leu, Ala) region that is not found in the other three families. The potential roles of the MEKHLA region in plants include participation in embryo patterning and auxin transport. However, the polymorphic nature of the interactions between HD-Zip III/IV family genes and the promoters of downstream genes adds complexity, thereby limiting the available research information [55] (Figure 1). Consequently, compared to other HD-Zip families, our understanding of the HD-Zip IV family remains limited, making the exploration of its gene functions an important research direction.

**Figure 2:**
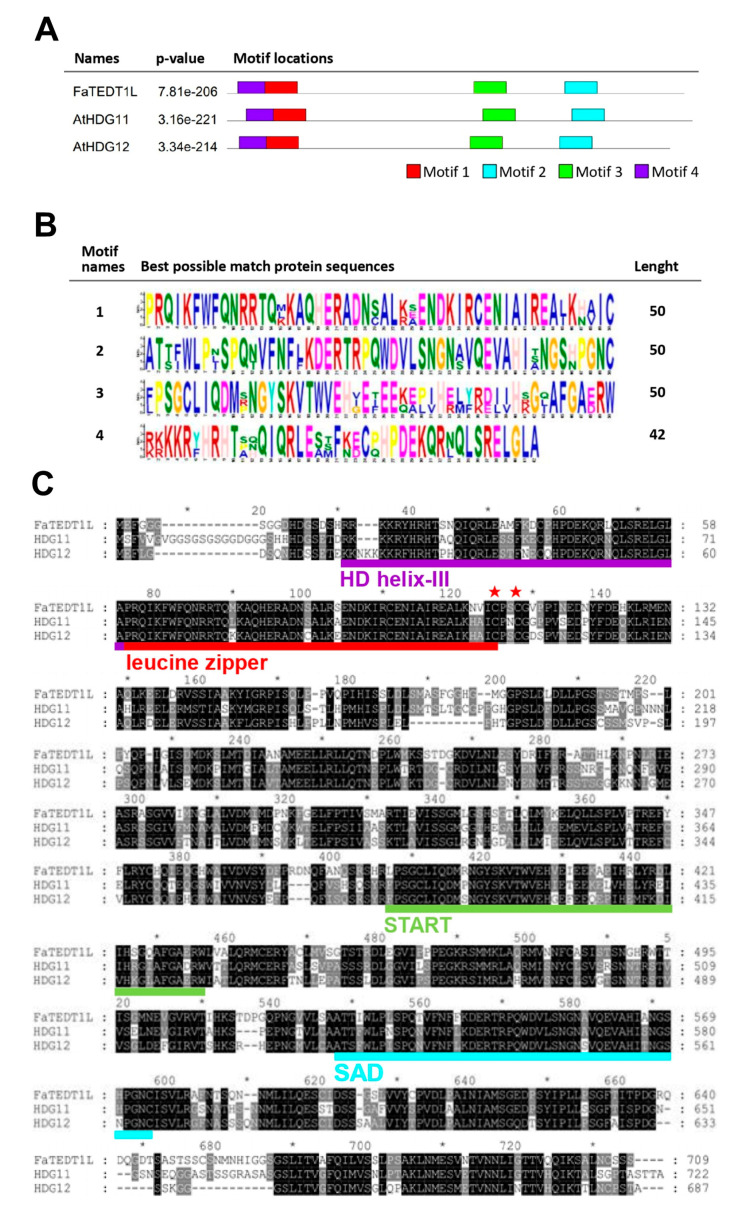
Functional domain prediction analysis of homologous proteins FaTEDT1L, AtHDG11, and AtHDG12 by using their amino acid sequences. (**A**) Using MEME analysis, the three gene products exhibit a similar distribution of functional domains, which resemble the HD-Zip group IV family. The expected *p*-values are listed next to each corresponding protein name. (**B**) Prediction of functional domain motifs 1 to 4 by using protein sequences. FaTEDT1L, AtHDG11, and AtHDG12 share conserved HD (motif 4), LZ (motif 1), START (motif 3), and SAD (motif 2) functional domains. (**C**) Amino acid sequence alignment of FaTEDT1L with *Arabidopsis* AtHDG11 and AtHDG12. As shown, amino acids with black background indicate complete amino acid identity among these three proteins, whereas amino acids with gray background show two of the three proteins share the same amino acid at that position. Amino acids lacking in one of the proteins are denoted with “-”. The predicted structural and functional domains are marked in two different colors, with Homeodomain helix-III (HD helix-III) shown in purple underline, Leucine zipper (LZ) in red underline, START domain in green underline, and SAD domain in blue underline, respectively. The red “★” symbol above the amino acids indicates the potential relevance to redox reactions.

To gain further insights into the potential functional regions of FaTEDT1L and the structural similarities among FaTEDT1L, AtHDG11, and AtHDG12, a comparative analysis of these protein sequences was performed. In addition, the functional domain prediction tool provided by the Multiple EM for Motif Elicitation (MEME) website was employed in order to identify the conserved functional motifs among these three proteins. Therefore, the amino acid sequences of FaTEDT1L, AtHDG11, and AtHDG12 were input into the MEME website with the settings for predicting four functional motifs, respectively. As a result, these proteins were found to exhibit similar distributions for functional motifs. Specifically, Motif 1 and Motif 4 were predicted to be located in the HD-ZIP domain, whereas Motif 3 belonged to the START domain, and Motif 2 was assigned to the SAD domain (Figure 2A). The predicted sequences for these four Motifs are shown in Figure 2B. Moreover, the common structural features in the important protein functional regions of FaTEDT1L and the HD-ZIP gene clusters were identified through sequence alignment analysis. These features include a homeodomain (HD) at the N-terminus, which is responsible for DNA binding, a leucine zipper (LZ) involved in specific DNA recognition, and two cysteine (Cys) residues predicted to be associated with redox reactions (Figure 1 and Figure 2B,C). Notably, pairwise sequence alignment analysis revealed that FaTEDT1L shares 67.8% identity with the full-length amino acid sequence of AtHDG11, slightly higher than the 65.3% identity shared with AtHDG12. Thus, our results collectively confirm that the complete sequence of the *FaTEDT1L* gene amplified through RT-PCR from the octoploid cultivated strawberry indeed represents the homologous gene of *AtHDG11* from the *Arabidopsis* (Figure 2C).

### 2.3. Gene Expression Analysis of FaTEDT1L in Different Tissues of Octoploid Cultivated Strawberry

To elucidate the gene expression pattern of *FaTEDT1L* across diverse tissues in *F. × ananassa* Duchessne cv. *Toyonoka*, we extracted total RNA from different tissues, including young leaves, mature leaves, flower buds, open flowers, stems, and stolons. Subsequently, cDNA was synthesized by using gene-specific primers targeting *FaTEDT1L*. Semi-quantitative reverse transcription PCR (Semi-qRT-PCR) was conducted to assess the relative expression levels of *FaTEDT1L* in these tissues. In addition to using specific primers for *FaTEDT1L*, we employed gene-specific primers for *FaActin* as an internal control in the experiments (Table S1).

**Figure 3:**
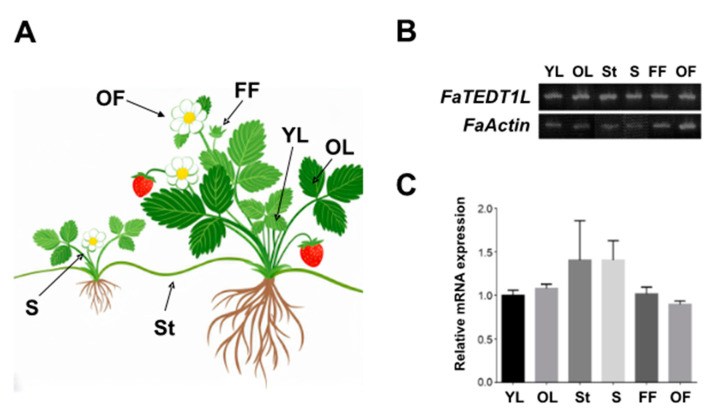
Gene expression analysis of *FaTEDT1L* in various tissues of octoploid strawberry (*F. × ananassa* Duchessne cv. *Toyonoka*) at the mRNA level. (**A**) Illustration of the sampled tissue locations, with young leaves labeled as YL, old leaves as OL, flower buds as FF, fully opened flowers as OF, runners as St, and stems as S, respectively. (**B**) Electrophoresis analysis of *FaTEDT1L* in different strawberry tissues after semi-quantitative reverse transcription PCR, with *FaActin* gene expression serving as the internal control. (**C**) Relative quantitative data analysis of gene expression levels from electrophoresis analysis. The value determined from YL tissue was set as 1 and relative values from other tissues were calculated from the electrophoresed gel shown in (**B**).

Sampling of octoploid cultivated strawberry tissues was mainly focused on the aerial parts of the plant, including young leaves, mature leaves, flower buds, open flowers, stems, and stolons (Figure 3A). Semi-qRT-PCR was performed using cDNA from each tissue, followed by agarose gel electrophoresis. The electrophoresed gel images were quantified by means of Image J software v11.0.10 to gain data representing the gene expression levels from each tissue. Both the gel analysis and quantification results revealed that *FaTEDT1L* gene expression was detected from all tissue samples (Figure 3B,C). Thus, our data are in close agreement with the previous findings for *AtEDT1*. Their work indicated that gene expression of *AtEDT1* was detected at the mRNA level in various aerial parts of *Arabidopsis*, including seedlings, roots, stems, leaves, flowers, and siliques [17,45]. It is of interest to note that other homologous genes of HD-ZIP group IV in different plant species may have distinct gene expression patterns. For example, *GaHDG11* of cotton exhibited similar gene expression patterns as those of *Arabidopsis* [53]. However, the homologous gene Glyma11g00570 of soybean was expressed only in flowers, whereas Glyma01g45070 was found to be expressed in young leaves, flowers, one-centimeter-long pods (one.cm.pod), and pod shells ten days after fertilization [56]. Interestingly, the homologous genes of HD-ZIP group IV identified in cucumber, all except *CsHDZIV5*, showed various degrees of gene expression in different tissues, including roots, stems, leaves, male flowers, fruits, and tendrils [57]. Taken together from the expression profiles of the above-mentioned genes in distinct plant tissues, it is evident that HD-ZIP group IV homologous genes from different plant species exhibit diverse gene expression patterns. Furthermore, these findings indicate that these homologous genes may not necessarily show tissue-specific expression.

### 2.4. Trans-Activation Activity of FaTEDT1L

**Figure 4:**
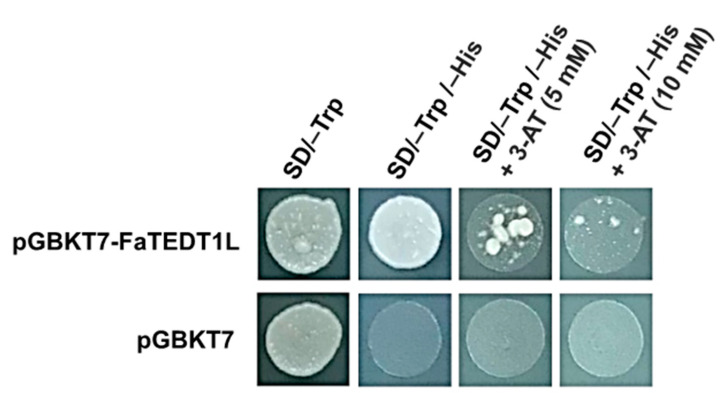
Transcriptional activation analysis of FaTEDT1L. After transforming pGBKT7-FaTEDT1L and pGBKT7 separately into yeast Y187 strain, the growth of transformed colonies on amino acid-deficient selection media was obtained. Plasmid pGBKT7-FaTEDT1L represents the recombinant vector containing the full-length *FaTEDT1L* gene fragment inserted into the pGBKT7 vector, while pGBKT7 serves as a negative control. SD/–Trp and SD/–Trp/–His show solid selection media lacking amino acid Tryptophan and both Tryptophan and Histidine, respectively; 3-AT is used to suppress the background expression of the *His3* reporter gene in the AH109 cells.

Transcription factors are known for their functional domains, particularly the DNA binding domain (DNA-BD), which enables them to bind to specific promoter regions of downstream genes. Subsequently, they exert their transcriptional activation function through the activation domain (AD), thereby influencing the expression of downstream genes at the transcriptional level (Figure 4). To investigate whether FaTEDT1L possesses the ability to activate downstream genes, a yeast one-hybrid assay was conducted. In this assay, a recombinant vector carrying the full-length *FaTEDT1L* (Full-length cDNA, FL) was introduced into yeast cells. A pGBKT7 vector was introduced into yeast cells as a negative control.

Firstly, both the empty pGBKT7 vector and the recombinant plasmid containing the full-length *FaTEDT1L* were transformed into the yeast AH109 strain. Subsequently, the transformed yeast cells were cultivated on solid selection media, including SD/–Trp, SD/–Trp/–His, and SD/–Trp/–His + 3-amino-1,2,4-triazole (3-AT) (5 mM and 10 mM, respectively). Our results showed that both the yeast cells carrying the pGBKT7 empty vector and those containing the recombinant plasmid (pGBKT7-FaTEDT1L) were able to grow colonies on the SD/–Trp selection medium. These data therefore indicated that transformation was successful, and the yeast colonies were able to grow normally on the selection medium lacking tryptophan (–Trp), which is a selection marker on the pGBKT7 vector (Figure 4). Next, the transformed colonies from pGBKT7-FaTEDT1L and pGBKT7 plates were picked and cultured in liquid SD/–Trp medium, respectively. The corresponding yeast cell suspension was then spotted onto solid SD/–Trp/–His selection medium. As shown, the results clearly indicated that the yeast cells transformed with the empty pGBKT7 vector did not form colonies on SD/–Trp/–His selection medium, thereby suggesting the empty pGBKT7 vector lacked any gene encoding a transcription factor and thus served as a negative control. On the other hand, yeast cells transformed with pGBKT7-FaTEDT1L indeed formed colonies on SD/–Trp/–His selection medium, demonstrating that the full-length *FaTEDT1L* gene product possesses transcription factor characteristics and is able to activate the downstream *His3* reporter gene expression (Figure 4).

Due to the phenomenon of “leaky expression” of the *His3* reporter gene on the pGBKT7 vector in AH109 cells, the yeast cell suspension was spotted onto solid SD/–Trp/–His medium in the presence of 3-AT at 5 mM and 10 mM, respectively. The leaky *HIS3* expression of one-hybrid pGBKT7 vector is first used to help construct *HIS3* reporter strains and later is controlled by including 3-aminotriazole (3-AT) in the medium to suppress the background yeast growth. Our data showed that yeast cells transformed with the empty pGBKT7 vector did not form colonies, while yeast cells transformed with pGBKT7-FaTEDT1L did form colonies on the SD/–Trp/–His + 3-AT solid selection medium. In addition, the number of colonies formed decreased as the concentration of 3-AT increased, indicating that 3-AT successfully suppressed the basal expression of *His3* within the yeast cells (Figure 4). The above-mentioned results thus exhibit that FaTEDT1L in octoploid cultivated strawberries, possibly along with other transcription factors, is involved in regulating the expression of downstream genes.

### 2.5. Establishment and Molecular Confirmation of Arabidopsis Transgenic Plants Overexpressing FaTEDT1L

**Figure 5:**
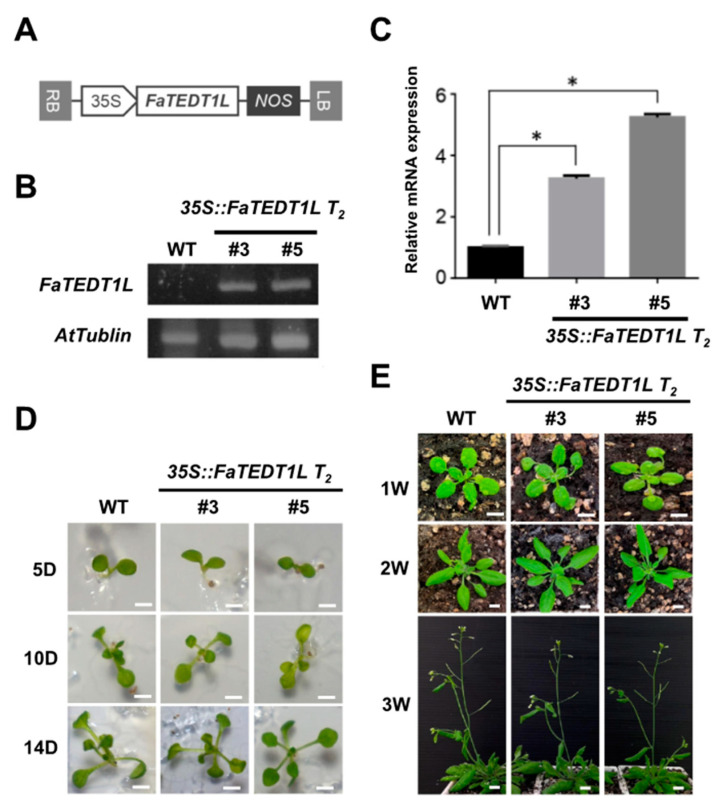
Establishment and molecular validation of *Arabidopsis* transgenic plants overexpressing *FaTEDT1L* in a wild-type (WT) background. Visual analysis of the characteristics of *35S::FaTEDT1L* T_2_ transgenic plants and WT plants at various growth stages. (**A**) Structural diagram of the T-DNA region in the recombinant plasmid, with the CaMV 35S promoter labeled as “35S (35S promoter)”, the terminator as “NOS (nopaline synthase terminator)”, and the T-DNA region’s left and right borders as “RB (Right border)” and “LB (Left border)”, respectively. (**B**) Gene expression analysis of *FaTEDT1L* at the mRNA level in *35S::FaTEDT1L* T2 transgenic plants and the WT, analyzed after semi-quantitative reverse transcription PCR. The *AtTubulin* (*AtTUB*) gene expression serves as the internal control. (**C**) Relative quantitative analysis of gene expression levels with values determined in the WT set as 1. Values from various transgenic plants are converted into relative ratios (*n* = 3, * *p* < 0.05). (**D**) Images were captured at five days (5D), ten days (10D), and fourteen days (14D) while growing on 1/2 MS medium. Bar = 2 mm. (**E**) Additional photographs were taken one week (1W), two weeks (2W), and three weeks (3W) after transplantation from the medium. Bar = 0.5 cm.

In order to explore the function of the *FaTEDT1L* gene in plants responsive to osmotic stress, we conducted a functional analysis of *FaTEDT1L* by using the *Arabidopsis* transformation platform. Following the gain-of-function strategy, the full-length *FaTEDT1L* gene was inserted into the pCambia1390/35S vector, thereby generating a pCambia1390/35S-FaTEDT1L recombinant vector (Figure 5A). Subsequently, this recombinant vector was introduced into the *Agrobacterium* strain GV3101. The floral dipping method was then employed as an *Agrobacterium*-mediated transformation, and transgenic *Arabidopsis* plants overexpressing *FaTEDT1L* were established. It was anticipated that these transgenic plants would exhibit abundant expression of *FaTEDT1L* at the transcriptional level driven by the Cauliflower mosaic virus (CaMV) 35S promoter, labeled as *35S::FaTEDT1L*. After transformation, T_1_ transgenic plants (*35S::FaTEDT1L T_1_*) with resistance to antibiotics Hygromycin (50 μg/mL) and Myron (30 μg/mL) were collected and transplanted for cultivation until they reached the flowering stage. T_2_ seeds were then produced by self-pollination of the T_1_ plants, and two transgenic lines were selected and named #3 and #5 (*35S::FaTEDT1L T_2_ #3* and *#5*), respectively. These two lines were further analyzed as molecular and/or transcriptional evidence in order to reveal the abundant expression of the *FaTEDT1L* in these transgenic plants.

Total RNA from the leaves of the #3 and #5 transgenic plants was extracted, respectively. Reverse transcription was conducted to synthesize cDNA, followed by a Semi-qRT-PCR reaction. Gel electrophoresis was performed to confirm the expression of *FaTEDT1L* at the transcriptional level in these transgenic plants. We used the wild-type (WT) without transformation as the negative control and the expression of the *AtTublin* gene as an internal control (Table S1). Our results showed that both *35S::FaTEDT1L T_2_ #3* and *35S::FaTEDT1L T_2_ #5* transgenic plants expressed the expected fragment size corresponding to the *FaTEDT1L* transgene via Semi-qRT-PCR analysis, while no product was detected from the WT plants (Figure 5B). The quantified data indicated that the expression level of the *FaTEDT1L* gene in *35S::FaTEDT1L T_2_ #3* and *35S::FaTEDT1L T_2_ #5* transgenic plants was significantly higher than that of the WT plants (Figure 5C). These above-mentioned experimental data thus demonstrated that the recombinant vector successfully delivered the *FaTEDT1L* transgene into different transgenic plants through *Agrobacterium* infection, leading to its stable overexpression in *35S::FaTEDT1L T_2_* transgenic plants.

Following the verification at the transcriptional level, we analyzed the phenotypic characteristics and morphology of the *35S::FaTEDT1L T_2_* transgenic plants. It was of interest to note that there were no significant differences in the physical appearance of the transgenic plants compared to that of the WT during the 14-day growth period in the growth chamber after seed germination (Figure 5D) or the subsequent three-week watering management in the greenhouse after transplantation (Figure 5E). These results were clearly shown by a bar indicator in the figure (Bar = 2 mm in Figure 5D; Bar = 0.5 cm in Figure 5E), although we did not actually measure different parts of the plants with a ruler. Thus, our results indicate that the overexpression of the *FaTEDT1L* gene in *Arabidopsis* with WT background does not affect plant growth, including seed germination, leaf development, flower structure, and silique formation at various developmental stages.

### 2.6. 35S::FaTEDT1L Transgenic Arabidopsis Plants Exhibited Enhanced Tolerance to Osmotic Stress

In order to further investigate the potential role of the strawberry gene *FaTEDT1L* in plant tolerance to osmotic stress, we conducted two types of stress tests on both *35S::FaTEDT1L* T_2_ transgenic plants and WT plants, one on culture medium and the other after transplantation into potting soil.

**Figure 6:**
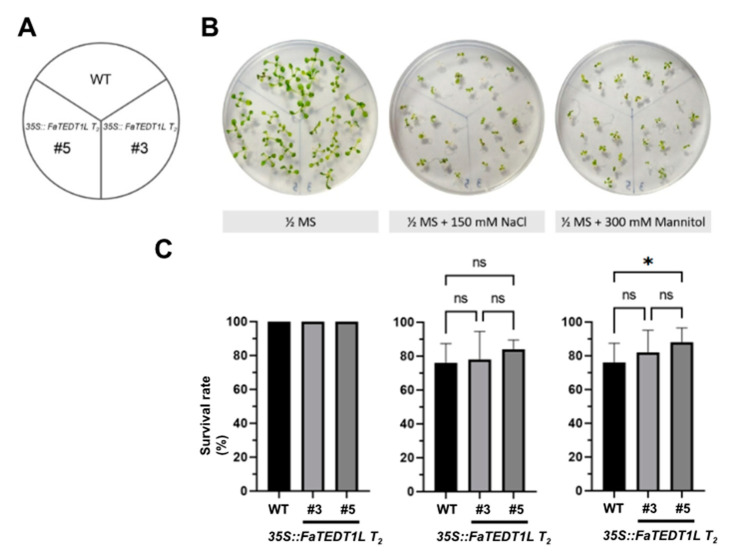
Comparing survival rates between *35S::FaTEDT1L* T_2_ transgenic plants and WT plants on 1/2 MS medium (1/2 MS), salt stress medium (1/2 MS + 150 mM NaCl), and osmotic stress medium (1/2 MS + 300 mM Mannitol), respectively. (**A**) Diagram illustrating the partition of different plants on culture plates. (**B**) Images of phenotypic characteristics of plants after 9 days on distinct stress plates. (**C**) The number of surviving plants on each plate was calculated, and subsequent ANOVA analysis was performed (*n* = 5, * *p* < 0.05). “ns” indicates no significant differences.

For the stress test on culture medium, *35S::FaTEDT1L* T_2_ seeds and WT seeds were sterilized and aseptically sown on 1/2 MS solid medium, respectively. After 5 days of growth under long-day conditions in the growth chamber, we selected unvaryingly germinated seedlings and individually transferred them into three types of media: 1/2 MS solid medium as a control, 1/2 MS with 150 mM NaCl representing high salinity stress, and 1/2 MS with 300 mM D-Mannitol indicating low water potential stress (Figure 6A). These plants were then kept in the growth chamber for another 14 days, during which we recorded their physical appearance as well as calculating the survival rate under each condition. The survival rates were determined as described in the Materials and Methods section. Our results revealed the average survival rates for WT, *35S::FaTEDT1L T_2_ #3*, and *35S::FaTEDT1L T_2_ #5* on 1/2 MS solid medium were all 100%, indicating no initial growth difference among these groups (Figure 6B,C). Under high salinity stress, the average survival rates were 76%, 78%, and 84% for the above-mentioned samples, respectively, also showing no significant difference among these three groups. In addition, there is no significant difference between transgenic plants #3 and #5 in terms of survival rates under normal or osmotic stress conditions (Figure 6C). However, under osmotic stress, the average survival rates were 76%, 82%, and 88% for WT, *35S::FaTEDT1L T_2_ #3*, and *35S::FaTEDT1L T_2_ #5*, respectively, displaying significant differences between WT and *35S::FaTEDT1L T_2_ #5* (*p* < 0.05) (Figure 6B,C).

**Figure 7:**
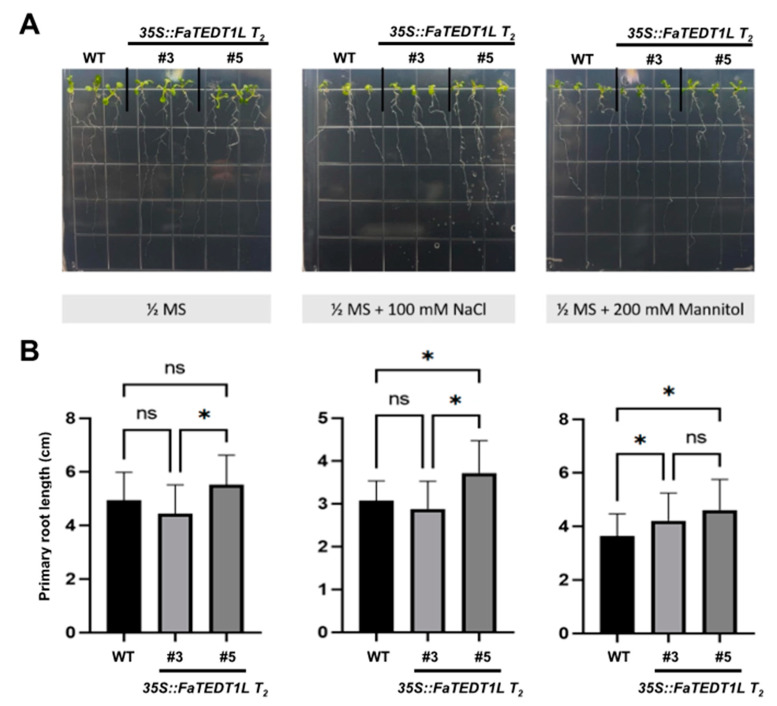
Root growth characteristics of *35S::FaTEDT1L* T_2_ transgenic plants (#3 and #5) and wild-type (WT) plants on 1/2 MS medium (1/2 MS), salt stress medium (1/2 MS + 100 mM NaCl), and osmotic stress medium (1/2 MS + 200 mM Mannitol), respectively. (**A**) Images of the root phenotypic characteristics of plants after 5 days on normal or different stress plates. (**B**) Measurement of the main root length of the plants from each plate and subsequent statistical analysis was conducted (*n* = 21, * *p* < 0.05). “ns” indicates no significant differences.

In addition, we examined the root lengths of the plants under each stress treatment compared to that of the WT. Briefly, each uniformly germinated seedling was transferred to 1/2 MS solid medium, 1/2 MS with 100 mM NaCl, or 1/2 MS with 200 mM D-Mannitol in order to simulate different stress conditions. After 10 days of growth in the growth chamber under long-day conditions, the main root length of the plants was measured for every group. Our data revealed that NaCl treatment caused a reduction in the root lengths of all groups (Figure 7A). The average root length on 1/2 MS solid medium was 4.93 ± 1.03 cm, 4.43 ± 1.08 cm, and 5.51 ± 1.13 cm for WT, *35S::FaTEDT1L T_2_ #3*, and *35S:: FaTEDT1L T_2_ #5*, respectively. A significant difference between *35S::FaTEDT1L T_2_ #3* and *#5* was detected, specifying that the higher overexpression of *FaTEDT1L* in transgenic plant #5 enhanced root growth under normal conditions. Notably, the average root length was 3.07 ± 0.46 cm, 2.88 ± 0.65 cm, and 3.71 ± 0.75 cm for WT, *35S::FaTEDT1L T_2_ #3*, and *35S:: FaTEDT1L T_2_ #5*, respectively, after NaCl treatment. Again, a significant difference was determined between *35S::FaTEDT1L T_2_ #3* and *#5*, as well as between WT and *35S::FaTEDT1L T_2_ #5*. Under osmotic stress, the average root length was 3.63 ± 0.83 cm, 4.20 ± 1.03 cm, and 4.60 ± 1.14 cm for the above-mentioned samples, respectively. A substantial difference between WT and *35S::FaTEDT1L T_2_ #3*, as well as between WT and *35S::FaTEDT1L T_2_ #5*, was noted (Figure 7B).

**Figure 8:**
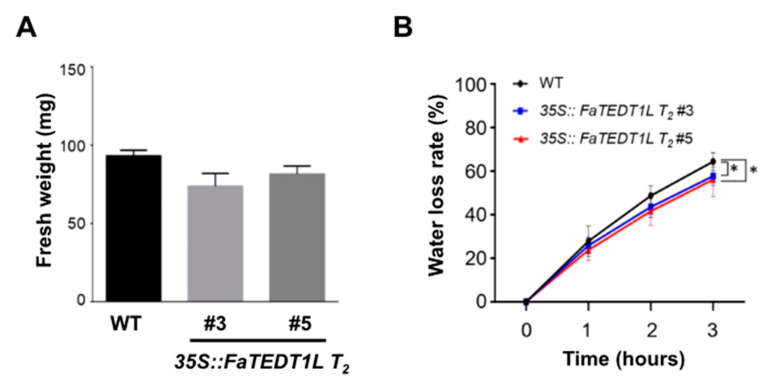
Analysis of plant water loss rates within *35S::FaTEDT1L* T_2_ transgenic plants and wild-type (WT) plants, respectively. (**A**) Comparison of the fresh weight of the above-ground parts of wild-type (WT), *35S::FaTEDT1L T_2_ #3* and *#5* transgenic plants grown on 1/2 MS medium under long-day conditions for 14 days (at 0 h of drought treatment) (*n* = 12, * *p* < 0.05). (**B**) Comparison of the water loss rates of the detached leaves of wild-type (WT), *35S::FaTEDT1L T_2_ #3* and *#5* transgenic plants after 1-, 2-, and 3-h drought treatment, respectively (*n* > 25, * *p* < 0.05).

In order to explore the transgenic plants in response to drought stress, we assessed the water loss within the plants as a direct factor for drought tolerance. Firstly, seeds of *35S::FaTEDT1L T_2_* and WT were germinated on 1/2 MS solid medium and subsequently grown in the growth chamber for 14 days. The above-ground parts of the plants were then weighed at time 0 (before drought treatment) and weighed every hour for a total of three hours (during drought treatment). The water loss rate was calculated based on the changes in fresh weights. Our results showed no significant difference in fresh weight among these three groups at time 0 (Figure 8A). However, as the drought treatment progressed, the water loss rate in the WT substantially exceeded that of the other two groups. At the end of the 3rd hour, the average water loss rates were 64%, 57.1%, and 55.6% for WT, *35S::FaTEDT1L T2 #3*, and *35S::FaTEDT1L T_2_ #5*, respectively. A significant difference between WT and *35S::FaTEDT1L T_2_ #3*, as well as between WT and *35S::FaTEDT1L T_2_ #5*, was determined (Figure 8B). Thus, these data revealed that the overexpression of *FaTEDT1L* in transgenic *Arabidopsis* plants could slow down the rate of water loss and consequently improve drought tolerance, thereby supporting the notion that reducing water loss is a critical factor in enhancing plant drought resistance.

**Figure 9:**
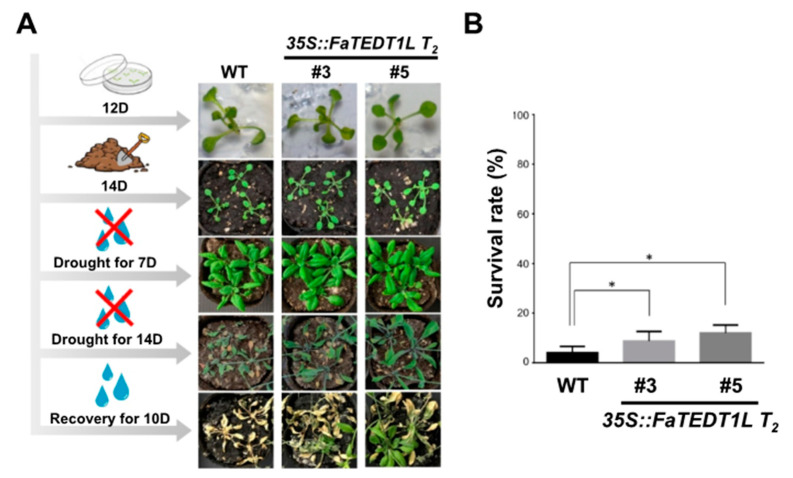
Drought stress tests and phenotypic analysis of *35S::FaTEDT1L* T_2_ transgenic plants and wild-type (WT) plants following transplantation. (**A**) Phenotypic images of plants after being transplanted 14 days of those grown on 1/2 MS medium for 12 days under long-day conditions and, thereafter, undergoing drought stress treatment. The timeline for drought stress treatment after transplanting is shown on the left of the images, 7 days (7D) and 14 days (14D), respectively. Ten days of re-watering (recovery for 10D) is indicated. (**B**) Statistical graph depicting the survival rates of WT and *35S::FaTEDT1L* T_2_ transgenic plants (#3 and #5) ten days after rehydration following drought stress treatment, respectively (*n* = 24, * *p* < 0.05).

Finally, we conducted drought tests on both *35S::FaTEDT1L T_2_* transgenic and WT plants after transplantation in order to perceive their growth under drought stress. Therefore, after 14 days of growth on 1/2 MS solid medium in the growth chamber under long-day conditions, the plants were transplanted into potting soil. Drought stress was then simulated by withholding water for either one week or two weeks. Following 7 or 14 days of drought treatment, photographs were taken, and a subsequent 10-day period of re-watering was carried out to assess the recovery capability of each plant. The survival rate was calculated according to the physical appearance of the plants. Our results showed no significant difference in terms of the physical appearance among these three groups, including those grown on the culture medium for 14 days, transplanted into potting soil, or transplanted into potting soil and subjected to one week of drought stress (Figure 9A). It is of interest to note that all groups showed wilting symptoms, with the plants appearing dark green and not yet turning brown by the third week of drought treatment. Following 10 days of re-watering, however, a slight difference in physical appearance was observed among these three groups. The WT exhibited more severe browning and withering, while some plants in *35S::FaTEDT1L T_2_ #3* and *35S:: FaTEDT1L T_2_ #5* groups showed signs of recovery due to rehydration (Figure 9A). The statistical data on the survival rates after the drought and re-watering tests indicated that the average recovery rates of WT, *35S::FaTEDT1L T_2_ #3*, and *35S::FaTEDT1L T_2_ #5* were 4%, 8.7%, and 12%, respectively. A significant difference was determined not only between WT and *35S::FaTEDT1L T2 #3* but also between WT and *35S::FaTEDT1L T_2_ #5* (Figure 9B). Therefore, our results provide evidence that the gene function of *FaTEDT1L* from strawberries is closely involved in the improved drought tolerance for plants.

### 2.7. Differential Expression Levels of Osmotic Stress-Regulated Pathway Genes in 35S::FaTEDT1L Transgenic Plants 

When plants encounter environmental adversity, they not only undergo physiological changes but also initiate a series of gene regulatory pathways in order to adapt to the challenges caused by adversity. These regulatory pathways involve numerous genes associated with stress responses. Building upon our previous research findings demonstrating that FaTEDT1L enhances the tolerance of transgenic plants to osmotic stress (Figure 6B,C), specific primer pairs for genes related to osmotic stress regulatory pathways were designed. These primers were then used to conduct molecular-level assessments between the WT and *35S::FaTEDT1L T_2_* transgenic plants, i.e., whether differential gene expression between these two under normal and stress conditions was determined. The targeted osmotic stress-related genes, including *SOS1* (*Salt overly sensitive 1*), *SOS2* (*Salt overly sensitive 2*), *SOS3* (Salt *overly sensitive 3*), *P5C1* (*Δ1-pyrroline-5-carboxylate synthetase 1*), *P5C2* (*Δ1-pyrroline-5-carboxylate synthetase 2*), *AREB1* (*abscisic acid-responsive element binding protein 1*), and *GSTU5* (*GST class tau 5*) were analyzed [58,59,60,61,62,63,64,65].

To simulate high salinity stress, 1/2 MS medium containing 150 mM NaCl was prepared. Subsequently, 14-day-old *35S::FaTEDT1L T_2_ #3*, *35S::FaTEDT1L T_2_ #5*, and WT seedlings grown on 1/2 MS medium were selected, respectively. These seedlings were immersed separately in 1/2 MS containing NaCl liquid medium for 1 h or 3 h as experimental groups, while seedlings not immersed (0 h) were served as the control groups. Following stress treatment, total RNA was extracted from the seedlings of each group. Reverse transcription reactions were then performed to synthesize their respective cDNA, and subsequent qPCR analysis was conducted by using gene-specific primer sets related to stress responses (Table S1).

**Figure 10:**
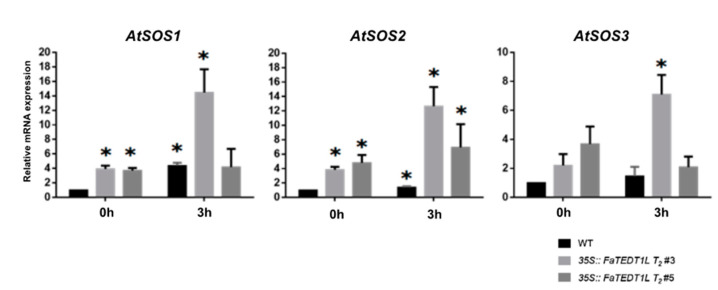
Gene expression analysis at the transcriptional level of *AtSOS1*, *AtSOS2,* and *AtSOS3* genes, related to osmotic stress response, was conducted through Real-Time qPCR in *35S::FaTEDT1L* T_2_ transgenic plants (#3 and #5) and wild-type (WT), respectively. The values detected in the untreated WT plants were set as 1, and the values determined in the untreated transgenic plants and different stress-treated plants were converted into relative ratios (*n* = 3, * *p* < 0.05).

The qPCR results of samples treated with 150 mM NaCl for 3h showed the upregulated expression of SOS1, SOS2, and SOS3 genes in *35S::FaTEDT1L T_2_ #3* and *35S::FaTEDT1L T_2_ #5* compared to the WT. Interestingly, substantial differences were determined in the expression of all three *SOS* genes between WT and *35S::FaTEDT1L T_2_ #3*. In addition, significantly differential *SOS2* gene expression between WT and *35S:: FaTEDT1L T_2_ #5* was noticed (Figure 10). Indeed, the expression of *SOS1*, *SOS2*, and *SOS3* was downregulated, and in others, it was upregulated. Although we do not have a firm answer for that observation so far, our explanation is that *SOS* gene expression in response to stress may occur at different developmental stages, resulting in down- or upregulation at different time points following stress treatment. Upregulated *P5CS1* and *P5CS2* gene expression in *35S::FaTEDT1L T_2_ #3* and *#5* compared to WT after 3 h salt treatment was noted. A significant difference in *P5CS2* gene expression was determined between WT and *35S::FaTEDT1L T_2_ #3* (Figure S4). On the other hand, the expression levels of ABA synthesis-related genes *AREB1* and *GSTU5* did not show a substantial increase in *35S::FaTEDT1L T_2_ #3* and *#5* transgenic plants (Figure S4). Here, we speculate that the phenomena we observed may be related to the detection timing and/or the involvement of other oxidative-reductive mechanisms in clearing free radicals, such as superoxide dismutase (SOD), catalase (CAT), peroxidase (POD), and others. Our results are in agreement with the previous notion that the Salt Overly Sensitive (SOS) signaling pathway is one of the primary defense mechanisms in plants in response to salt stress. It is inferred that *35S::FaTEDT1L T_2_ #3* and *#5* transgenic plants may effectively extrude sodium ions from the cells via the SOS complex composed of *SOS1*, *SOS2*, and *SOS3* genes, mediating cell signaling under salt stress in order to maintain ion homeostasis and thus reduce the damage to protoplasts [62,63]. Additionally, through the action of the enzymes *P5CS1* and *P5CS2*, these transgenic plants may synthesize proline, which in turn increases the accumulation of compatible solutes within the cells, thereby enhancing the cell’s water potential and further conferring the ability to withstand osmotic stress [64,65].

To determine whether the promoter regions of the *SOS* genes (*SOS1*, *SOS2*, *SOS3*) and the *P5CS2* structural gene contain cis-acting elements that can be bound by FaTEDT1L, we performed The Plant Promoter Analysis Navigator 4.0 [PlantPAN 4.0, http://plantpan.itps.ncku.edu.tw/plantpan4/gene_info.php (accessed on 6 September 2024)] [66]. Through this analysis, whether the promoter regions of these four genes have binding sites for the HD-ZIP domain of the transcription factors, such as AtEDT1, was predicted in *Arabidopsis*. The predicted results revealed the binding sequences (a(t/a/c)(t/a)TTAATg(g/t) and ccATTAAttta) located within 1 kb upstream of the Transcription Start Site (TSS) that can be recognized by the AtEDT1 transcription factor (Figure S5). These data thus suggest that the homologous transcription factor FaTEDT1L may also have the potential to bind to the promoter regions of *SOS1*, *SOS2*, *SOS3*, and *P5CS2*, leading to the regulation of these genes expression in transgenic plants. These findings further corroborate and support our qPCR results.

## 3. Materials and Methods

### 3.1. Plant Materials and Growth Conditions

The plant materials used in this study included the octoploid cultivated strawberry (*F*. *× ananassa* Duchesne cv. *Toyonoka*) and *Arabidopsis thaliana*. The octoploid strawberries were purchased from the Taiwan Seed Improvement and Propagation Station, MOA. Taichung, Taiwan. These strawberry seedlings were acclimatized after plant tissue culture and cultivated in a growth chamber set at a temperature of 23–25 °C, with a relative humidity of approximately 60–80%, under long-day conditions (16 h of light/8 h of darkness). All *Arabidopsis* plants used in this study were of the Columbia (Col-0) ecotype obtained from the Arabidopsis Biological Resource Center (ABRC, Ohio State University, Columbus, OH, USA). For in vitro growth, seeds were surface-sterilized and sown on 1/2 × Murashige and Skoog (MS) agar medium (PhytoTech, KS, USA) containing 2% (*w*/*v*) sucrose in Petri dishes and kept in a growth chamber at 22 °C under a long-day cycle (16 h of light/8 h of darkness). After 14 days of growth under these conditions, the *Arabidopsis* seedlings were transplanted into soil for further cultivation under the same long-day conditions.

### 3.2. Cloning of the cDNA for FaTEDT1L Genes from the Octoploid Cultivated Strawberry

Based on the transcriptome dataset of the octoploid cultivated strawberry (*F*. *× ananassa* Duchesne cv. *Toyonoka*) [53] and the original sequencing data available from the Sequence Read Archive (SRA) at the National Center for Biotechnology Information (NCBI) under accession numbers SRX1895539 and SRX1895540 for the OF and DU transcriptome datasets, respectively, the CLCbio program (QIAGEN CLC Genomics Workbench 20) was utilized to reconstruct gene sequences. These sequences were used as a search database in order to identify potential gene candidates analogous to AtEDT1, which is associated with drought tolerance. The FaTEDT1L (*F*. *× ananassa* Duchesne cv. *Toyonoka* Enhanced Drought Tolerance 1-Like) gene, homologous to AtEDT1/HDG11, was then identified. The full-length *FaTEDT1L* gene was amplified from Toyonoka strawberry cDNA via PCR by using gene-specific primer sets (Table S1). These primers were designed to facilitate the cloning process, utilizing Platinum^®^ Taq DNA polymerase (Invitrogen, Carlsbad, CA, USA). The resulting PCR product was purified by means of a BIOKIT purification column, following the manufacturer’s protocol. The purified product was subsequently cloned into the pGEM-T Easy vector (Promega, Madison, WI, USA) and sequenced to verify the DNA sequences of the selected clones using internal gene-specific primers for *FaTEDT1L* (Table S1).

### 3.3. Sequence Alignment and Phylogenetic Analyses

The deduced amino acid sequences of FaTEDT1L were aligned with 48 known *Arabidopsis* protein sequences containing the HD-ZIP domain. These gene products include those from the HD-Zip I (17 members), HD-Zip II (10 members), HD-Zip III (5 members), and HD-Zip IV (16 members) families, obtained from the NCBI database [http://www.ncbi.nlm.nih.gov/ (accessed on 12 February 2023)]. Bootstrap values were calculated using ClustalX version 1.83 (European Bioinformatics Institute, Hinxton, Cambridge, UK) [67,68]. Genetic distances were computed with the Kimura 2-parameter model [69], and a phylogenetic tree was constructed via the neighbor-joining (NJ) method in MEGA X [54]. The numbers at the tree nodes represent bootstrap values from 1000 replicates.

### 3.4. RT-PCR and Real-Time Quantitative RT-PCR (qRT-PCR)

The expression level of the *FaTEDT1L* gene in the octoploid strawberry *F. × ananassa* Duchessne cv. Toyonoka was measured using RT-PCR assays. Total RNA was extracted from young leaves, mature leaves, flower buds, open flowers, stems, and stolons, respectively, by using a cetyl trimethylammonium bromide-based buffer described previously [53]. First-strand cDNA was synthesized via the GoScript™ reverse transcription system (Promega, Madison, WI, USA) following the manufacturer’s instructions. The cDNA samples were amplified with the primers FaTEDT1L-1F and FaTEDT1L-1R (Table S1) under the following conditions: denaturation at 94 °C for 3 min, followed by 30 cycles of 94 °C for 30 s, 58 °C for 45 s, and 72 °C for 2 min, and a final extension at 72 °C for 7 min. The *Actin* gene was used as an internal control, with primers FaActin-F and FaActin-R (Table S1). Both PCR and RT-PCR products were separated on 1% agarose gels stained with ethidium bromide and visualized via an ultraviolet gel imaging system. For qPCR, 1 µL of the cDNA sample (diluted 10×) from the RT reactions was employed in a reaction as follows: 95 °C for 5 min, followed by 35 cycles of 95 °C for 20 s, 60 °C for 30 s, and 72 °C for 30 s. The qRT-PCR reactions were performed by means of the Roche LightCycler^®^-480 Real-Time PCR system with the KAPA SYBR FAST Universal qPCR Kit (KAPA BIOSYSTEMS) according to the manufacturer’s instructions. Primer sequences for osmotic stress-related genes and the internal control gene are listed in Table S1. Expression levels of all osmotic stress-related genes were quantified by using the 2^−ΔΔCT^ method, with three biological replicates and three technical replicates. Statistical significance was determined via ANOVA or the Student’s *t*-test.

### 3.5. Transactivation Analysis in Yeast Cells

The full-length cDNA for *FaTEDT1L* was generated via RT-PCR reactions by using gene-specific primers. The amplified fragment was then cloned into the pGBKT7 plasmid, a vector containing a GAL4 DNA-binding domain (GAL4DB vector). Thereafter, yeast transformation and one-hybrid analyses were performed using the *Sacccharomyces cerevisiae* One-Step (SCOS) Transformation Kit (YB Biotech, New Taipei, Taiwan). Both the pGBKT7-FaTEDT1L recombinant construct and the empty pGBKT7 vector were separately transformed into the yeast strain AH109, cells comprising a *Histidine* reporter gene (*His3*). Interactions between DNA [upstream activating sequences (UASs) and TATA boxes of the *His3* reporter gene promoter region] and the protein (the GAL4DB target gene fusion protein) were assessed by the growth of yeast cells on selective synthetic defined medium lacking amino acids tryptophan and histidine (SD/-Trp/-His) as well as on the growth of control medium (SD/-Trp). Thus, DNA–protein interactions were ultimately confirmed through spotting assays, which were repeated at least three times.

### 3.6. Generation of Transgenic Plants

To ensure the correct orientation of insertion, the T-vector containing the full-length *FaTEDT1L* cDNA was screened by releasing the insert following *EcoRI* digestion. The confirmed insert was then sub-cloned from the pGEM-T easy vector (Promega) into the binary vector pCambia1390-35S (CAMBIA). The presence of the *35S::FaTEDT1L* construct in the pCambia1390-35S vector was verified by gene-specific PCR and restriction enzyme digestion analysis. The resulting constructs were subsequently introduced into *Agrobacterium tumefaciens* strain GV3101. Thereafter, the *35S::FaTEDT1L* construct was transformed into wild-type *Arabidopsis thaliana* Col-0 ecotype plants via the floral-dip method [70]. The harvested seeds were surface-sterilized with a 25% sodium hypochlorite solution. Transgenic plants comprising *35S::FaTEDT1L* were selected on solid 1/2 MS medium containing 30 µg/mL hygromycin. The plates were placed vertically in a growth chamber (CH-202, CHIN-HSIN, Yunlin, Taiwan) under long-day conditions (16-h light/8-h dark cycle), with a light intensity of 120 µmol m^−2^ s^−1^ and a temperature of 22 °C ± 2 °C, for 12 days before transplanting into soil. The ectopic expression of *FaTEDT1L* in the transgenic *Arabidopsis* lines was confirmed by RT-PCR reactions using gene-specific primers (Table S1). Digital images of the whole transgenic plants were obtained, and young seedlings of both wild-type and transgenic plants were observed under a dissecting microscope (Nikon, SMZ745T, Tokyo, Japan). All images were captured by means of a digital camera under light-field conditions.

### 3.7. Salt and Osmotic Stress Treatment in Arabidopsis 

Two transgenic lines from the T_2_ generation (*35S::FaTEDT1L T_2_ #3* and *#5*), which exhibited high levels of *FaTEDT1L* expression, along with a wild-type control, were selected for drought and salt stress treatments. All sterilized seeds were sown on 1/2 MS solid medium. After growing under long-day conditions for five days, uniformly germinated seedlings were chosen and individually transferred to three different media types: 1/2 MS with 150 mM NaCl for high salinity stress, 1/2 MS with 300 mM D-mannitol for low water potential stress, and 1/2 MS medium as a control. On day 14, photographs were taken, and the survival rates for each group were recorded. Survival rate (%) was calculated as the number of surviving seedlings divided by the total number of seedlings multiplied by 100%. Ten equally sprouted seedlings of each line were chosen from each stress treatment, and five replicates were performed. To assess the root lengths of the *35S::FaTEDT1L T_2_ #3* and *#5* transgenic lines following osmotic stress treatment, three unvaryingly burgeoned seedlings were selected after five days on 1/2 MS solid medium. These seedlings were then transferred to either 1/2 MS medium containing 100 mM NaCl, 200 mM D-mannitol, or a control 1/2 MS medium. After 10 days of growth under long-day conditions, the primary roots of each plant were photographed. The root lengths were measured by means of Image J software, and the data were statistically analyzed. The experiments were conducted with seven replicates. Water loss rates were assessed by using more than 25 seedlings, each from the wild-type, *35S::FaTEDT1L T_2_ #3*, and *#5* transgenic lines. Two-week-old plants grown on 1/2 MS solid medium were uprooted and immediately weighed. The plants were then placed on a plate on top of a laboratory bench and reweighed at the specified time intervals. The percentage of fresh weight loss was calculated relative to the initial weight of the plant. To conduct soil drought tests for both *35S::FaTEDT1L T_2_* transgenic and wild-type plants, 14-day-old seedlings grown on 1/2 MS solid medium under long-day conditions were transplanted into potting soil. Drought stress was simulated by withholding water for either one or two weeks. Following the drought treatment, the plants were re-watered for 10 days to evaluate their recovery ability. Photographs were taken, and survival rates were analyzed.

## 4. Conclusions

Previous studies have shown the multifunctionality of the *EDT1*/*HDG11* gene, which not only regulates trichome formation and morphological establishment in *Arabidopsis* but also plays a role in plant responses, particularly in non-biological stress situations such as osmotic stress, including drought and salinity [39]. Here, we identified the full-length sequences of the *FaTEDT1L* gene from the *F.* × *ananassa* Duchessne cv. *Toyonoka* transcriptome sequencing data. Following sequence confirmation, FaTEDT1L protein was subjected to phylogenetic and multiple sequence alignment analyses with 48 known *Arabidopsis* HD-ZIP gene family members, revealing that FaTEDDT1L from octoploid cultivated strawberry is a homolog of AtHDG11 within the subgroup IV of *Arabidopsis* HD-ZIP gene families. Furthermore, FaTEDT1L was shown to be a transcription factor with a functional activation domain for regulating the downstream gene expression via a yeast one-hybrid system. To further explore *FaTEDT1L* gene function, we generated transgenic *Arabidopsis* plants (*35S::FaTEDT1L*) with overexpression of the octoploid *FaTEDT1L* gene from *F.* × *ananassa* Duchessne cv. *Toyonoka*. Our data revealed that *FaTEDT1L* led to an increased tolerance to osmotic stress in *Arabidopsis* when this gene was ectopically overexpressed. Based on the results of various simulated stress tests, we inferred that *35S::FaTEDT1L* containing transgenic plants exhibit enhanced resistance to osmotic stress compared to the wild-type for several reasons. Firstly, they increase tolerance by extending the root growth, thereby expanding the area for water and nutrient absorption from the deeper soil layers. Secondly, the rate of water loss within the transgenic plants is reduced, as shown in this study. Lastly, FaTEDT1L positively regulates the expression of *SOS* genes (*SOS1*, *SOS2*, *SOS3*) as well as in part regulating the *P5CS2* gene expression, which in turn increases sodium ion transport out of the cells and leads to proline accumulation. Therefore, these above-mentioned results successfully enhance the drought resistance of transgenic plants and survival rate under osmotic stress conditions. Taken together, our data in this study suggest that *FaTEDT1L* is a functional gene candidate in *F.* × *ananassa* with the potential for future applications in strawberry breeding and increasing crop resilience in response to stress conditions in improving crop yield. In other words, scientists can easily screen and select strawberry lines with highly expressed *FaTEDT1L* instead of making GMOs (genetically modified organisms), which is the true novelty of this study.

## Data Availability

The original contributions presented in the study are included in the article/Supplementary Material, further inquiries can be directed to the corresponding author/s.

## Supplementary Materials

The following supporting information can be downloaded at: https://www.mdpi.com/article/10.3390/ijms251810091/s1.
