# Supplementary material for: FaTEDT1L of Octoploid Cultivated Strawberry Functions as a Transcriptional Activator and Enhances Abiotic Stress Tolerance in Transgenic Arabidopsis
Source: Int J Mol Sci. 2024 Sep 19;25(18):10091. doi: 10.3390/ijms251810091 (PMC11432484; doi:10.3390/ijms251810091)
Supplement: Supplementary file 1 [file ijms-25-10091-s001.zip › ijms-3191043-supplementary.pdf]

Supplementary Materials: *FaTEDT1L* of octoploid cultivated strawberry functions as a transcriptional activator and enhances stress tolerance in transgenic *Arabidopsis*

Ching-Ying Chu [1,†], Lee-Fong Lin [1,2,†], Shang-Chih Lai [3], Jui-Hung Yang [4], and Ming-Lun Chou [1,2,*]

Table S1. Primers used in this study.

Target gene	Forward primer (5' to 3')	Reverse primer (5' to 3')	Purpose
FaTEDT1L	ATGGAGTTTGGCGGTGGCTCCGGTGGA	TCAGGAACTACTACAATTCAAGGCAGA	RT-PCR
	GCCCCAATTCATCGACTTTATAGAGAT		sequencing
	GGCATTTCGGACATGGACAAGTCCCTC		sequencing
	ATCTCTATAAAGTCGATGAATTGGGGC		sequencing
	GAGGGACTTGTCCATGTCCGAAATGCC		sequencing
FaActin	ACTGGTATGGTCAAGGCCGGGTTT	ACACCATCACCAGAGTCGAGCACG	RT-PCR
AtTublin	CTCAAGAGGTTCTCAGCAGTA	TCACCTTCTTCATCCGCAGTT	RT-PCR
AtSOS1-qRT	TTCATCATCCTCACAATGGCTCTAA	CCCTCATCAAGCATCTCCCAGTA	Real-time qPCR
AtSOS2-qRT	GAACTCCGAACTATGTAG	TATCCAGCCAATATAACG	Real-time qPCR
AtSOS3-qRT	AGAGGAAGATAGAGATGTAAGC	ATTATGTATGTGAGATGGAGAGT	Real-time qPCR
AtP5CS1-qRT	CAAGATGAGATTACATTCG	GGTTATGATGACAGGAAT	Real-time qPCR
AtP5CS2-qRT	GGCTTACTATGAGACTATGT	ACTAAGTTGCTTCCTGAA	Real-time qPCR
AtAREB1-qPCR	TCACTCTCTCCGTCTCCTTAC	CATTCTCCTTTGCCTTCTCTCT	Real-time qPCR
AtGSTU5-qPCR	ATGGCTGAGAAAGAAGAAGTGAAGC	TTAAGAAGATCTCACTCTCTCTGCC	Real-time qPCR
AtActin-qRT	ATGCCCAGAAGTCTTGTTCC	TGCTCATACGGTCAGCGATA	Real-time qPCR

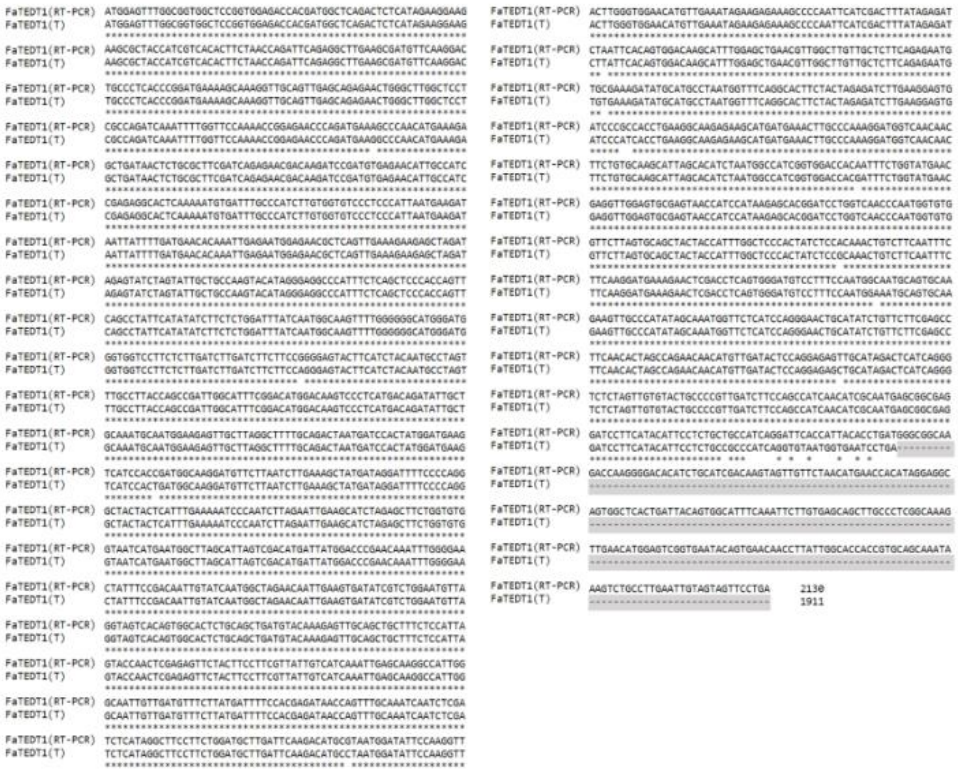

Figure S1. Gene sequences analysis of FaTEDT1 (RT-PCR) and FaTEDT1 (T). FaTEDT1 (RT-PCR) represents the gene sequence of *FaTEDT1* obtained from RT-PCR, while FaTEDT1 (T) indicates the gene sequence of *FaTEDT1* assembled from the *F.* × *ananassa* Duchessne cv. *Toyonoka* transcriptome database. Nucleotides shown with "*" exhibit complete matches between FaTEDT1 (RT-PCR) and FaTEDT1 (T). The gray areas demonstrate sequence information lacking in the transcriptome assembled [FaTEDT1 (T)], denoted with "-".

```
   1 atg gag ttt ggc ggt ggc tcc ggt gga gac cac gat ggc tca gac tct cat aga agg aag
   1  M   E   F   G   G   G   S   G   G   D   H   D   G   S   D   S   H   R   R   K
  61 aag cgc tac cat cgt cac act tct aac cag att cag agg ctt gaa gcg atg ttc aag gac
  21  K   R   Y   H   R   H   T   S   N   Q   I   Q   R   L   E   A   M   F   K   D
 121 tgc cct cac ccg gat gaa aag caa agg ttg cag ttg agc aga gaa ctg ggc ttg gct cct
  41  C   P   H   P   D   E   K   Q   R   L   Q   L   S   R   E   L   G   L   A   P
 181 cgc cag atc aaa ttt tgg ttc caa aac cgg aga acc cag atg aaa gcc caa cat gaa aga
  61  R   Q   I   K   F   W   F   Q   N   R   R   T   Q   M   K   A   Q   H   E   R
 241 gct gat aac tct gcg ctt cga tca gag aac gac aag atc cga tgt gag aac att gcc atc
  81  A   D   N   S   A   L   R   S   E   N   D   K   I   R   C   E   N   I   A   I
 301 cga gag gca ctc aaa aat gtg att tgc cca tct tgt ggt gtc cct ccc att aat gaa gat
 101  R   E   A   L   K   N   V   I   C   P   S   C   G   V   P   P   I   N   E   D
 361 aat tat ttt gat gaa cac aaa ttg aga atg gag aac gct cag ttg aaa gaa gag cta gat
 121  N   Y   F   D   E   H   K   L   R   M   E   N   A   Q   L   K   E   E   L   D
 421 aga gta tct agt att gct gcc aag tac ata ggg agg ccc att tct cag ctc cca cca gtt
 141  R   V   S   S   I   A   A   K   Y   I   G   R   P   I   S   Q   L   P   P   V
 481 cag cct att cat ata tct tct ctg gat tta tca atg gca agt ttt ggg ggg cat ggg atg
 161  Q   P   I   H   I   S   S   L   D   L   S   M   A   S   F   G   G   H   G   M
 541 ggt ggt cct tct ctt gat ctt gat ctt ctt ccg ggg agt act tca tct aca atg cct agt
 181  G   G   P   S   L   D   L   D   L   L   P   G   S   T   S   S   T   M   P   S
 601 ttg cct tac cag ccg att ggc att tcg gac atg gac aag tcc ctc atg aca gat att gct
 201  L   P   Y   Q   P   I   G   I   S   D   M   D   K   S   L   M   T   D   I   A
 661 gca aat gca atg gaa gag ttg ctt agg ctt ttg cag act aat gat cca cta tgg atg aag
 221  A   N   A   M   E   E   L   L   R   L   L   Q   T   N   D   P   L   W   M   K
 721 tca tcc acc gat ggc aag gat gtt ctt aat ctt gaa agc tat gat agg att ttc ccc agg
 241  S   S   T   D   G   K   D   V   L   N   L   E   S   Y   D   R   I   F   P   R
 781 gct act act cat ttg aaa aat ccc aat ctt aga att gaa gca tct aga gct tct ggt gtg
 261  A   T   T   H   L   K   N   P   N   L   R   I   E   A   S   R   A   S   G   V
 841 gta atc atg aat ggc tta gca tta gtc gac atg att atg gac ccg aac aaa ttt ggg gaa
 281  V   I   M   N   G   L   A   L   V   D   M   I   M   D   P   N   K   F   G   E
 901 cta ttt ccg aca att gta tca atg gct aga aca att gaa gtg ata tcg tct gga atg tta
 301  L   F   P   T   I   V   S   M   A   R   T   I   E   V   I   S   S   G   M   L
 961 ggt agt cac agt ggc act ctg cag ctg atg tac aaa gag ttg cag ctg ctt tct cca tta
 321  G   S   H   S   G   T   L   Q   L   M   Y   K   E   L   Q   L   L   S   P   L
1021 gta cca act cga gag ttc tac ttc ctt cgt tat tgt cat caa att gag caa ggc cat tgg
 341  V   P   T   R   E   F   Y   F   L   R   Y   C   H   Q   I   E   Q   G   H   W
1081 gca att gtt gat gtt tct tat gat ttt cca cga gat aac cag ttt gca aat caa tct cga
 361  A   I   V   D   V   S   Y   D   F   P   R   D   N   Q   F   A   N   Q   S   R
1141 tct cat agg ctt cct tct gga tgc ttg att caa gac atg cgt aat gga tat tcc aag gtt
 381  S   H   R   L   P   S   G   C   L   I   Q   D   M   R   N   G   Y   S   K   V
1201 act tgg gtg gaa cat gtt gaa ata gaa gag aaa gcc cca att cat cga ctt tat aga gat
 401  T   W   V   E   H   V   E   I   E   E   K   A   P   I   H   R   L   Y   R   D
1261 cta att cac agt gga caa gca ttt gga gct gaa cgt tgg ctt gtt gct ctt cag aga atg
 421  L   I   H   S   G   Q   A   F   G   A   E   R   W   L   V   A   L   Q   R   M
1321 tgc gaa aga tat gca tgc cta atg gtt tca ggc act tct act aga gat ctt gaa gga gtg
 441  C   E   R   Y   A   C   L   M   V   S   G   T   S   T   R   D   L   E   G   V
1381 atc ccg cca cct gaa ggc aag aga agc atg atg aaa ctt gcc caa agg atg gtc aac aac
 461  I   P   P   P   E   G   K   R   S   M   M   K   L   A   Q   R   M   V   N   N
1441 ttc tgt gca agc att agc aca tct aat ggc cat cgg tgg acc aca att tct ggt atg aac
 481  F   C   A   S   I   S   T   S   N   G   H   R   W   T   T   I   S   G   M   N
1501 gag gtt gga gtg cga gta acc atc cat aag agc acg gat cct ggt caa ccc aat ggt gtg
 501  E   V   G   V   R   V   T   I   H   K   S   T   D   P   G   Q   P   N   G   V
1561 gtt ctt agt gca gct act acc att tgg ctc cca cta tct cca caa act gtc ttc aat ttc
 521  V   L   S   A   A   T   T   I   W   L   P   L   S   P   Q   T   V   F   N   F
1621 ttc aag gat gaa aga act cga cct cag tgg gat gtc ctt tcc aat ggc aat gca gtg caa
 541  F   K   D   E   R   T   R   P   Q   W   D   V   L   S   N   G   N   A   V   Q
1681 gaa gtt gcc cat ata gca aat ggt tct cat cca ggg aac tgc ata tct gtt ctt cga gcc
 561  E   V   A   H   I   A   N   G   S   H   P   G   N   C   I   S   V   L   R   A
1741 ttc aac act agc cag aac aac atg ttg ata ctc cag gag agt tgc ata gac tca tca ggg
 581  F   N   T   S   Q   N   N   M   L   I   L   Q   E   S   C   I   D   S   S   G
1801 tct cta gtt gtg tac tgc ccc gtt gat ctt cca gcc atc aac atc gca atg agc ggc gag
 601  S   L   V   V   Y   C   P   V   D   L   P   A   I   N   I   A   M   S   G   E
1861 gat cct tca tac att cct ctg ctg cca tca gga ttc acc att aca cct gat ggg cgg caa
 621  D   P   S   Y   I   P   L   L   P   S   G   F   T   I   T   P   D   G   R   Q
1921 gac caa ggg gac aca tct gca tcg aca agt agt tgt tct aac atg aac cac ata gga ggc
 641  D   Q   G   D   T   S   A   S   T   S   S   C   S   N   M   N   H   I   G   G
1981 agt ggc tca ctg att aca gtg gca ttt caa att ctt gtg agc agc ttg ccc tcg gca aag
 661  S   G   S   L   I   T   V   A   F   Q   I   L   V   S   S   L   P   S   A   K
2041 ttg aac atg gag tcg gtg aat aca gtg aac aac ctt att ggc acc acc gtg cag caa ata
 681  L   N   M   E   S   V   N   T   V   N   N   L   I   G   T   T   V   Q   Q   I
2101 aag tct gcc ttg aat tgt agt agt tcc tga
 701  K   S   A   L   N   C   S   S   S   -
```

Figure S2. The complete cDNA sequences and their encoding amino acids of *FaTEDT1L* gene

```
FaTEDT1L  MEFGGGSGGDHDGSDSHRRKKRYHRHTSNQIQRLEAMFKDCPHPDEKQRLQLSRELGLAP
FvEDT1    MEFGGGSGGDHDGSDSHRRKKRYHRHTSNQIQRLEAMFKDCPHPDEKQRLQLSRELGLAP
          ************************************************************

FaTEDT1L  RQIKFWFQNRRTQMKAQHERADNSALRSENDKIRCENIAIREALKNVICPSCGVPPINED
FvEDT1    RQIKFWFQNRRTQMKAQHERADNSVLRSENDKIRCENIAIREALKNVICPSCGVPPINED
          ************************.***********************************

FaTEDT1L  NYFDEHKLRMENAQLKEELDRVSSIAAKYIGRPISQLPPVQPIHISSLDLSMASFGGHGM
FvEDT1    NYFDEHKLRMENAQLKEELDRVSSIAAKYIGRPISQLPPVQPIHISSLDLSMASFGGHGM
          ************************************************************

FaTEDT1L  GGPSLDLDLLPGSTSSTMPSLPYQPIGISDMDKSLMTDIAANAMEELLRLLQTNDPLWMK
FvEDT1    GGPSLDLDLLPGSTSSTMPSLPYQPIGISDMDKSLMTDIAANAMEELLRLLQTNDPLWMK
          ************************************************************

FaTEDT1L  SSTDGKDVLNLESYDRIFPRATTHLKNPNLRIEASRASGVVIMNGLALVDMIMDPNKFGE
FvEDT1    SSTDGKDVLNLESYDRIFPRATTHLKNPNLRIEASRASGVVIMNGLALVDMIMDPNKFGE
          ************************************************************

FaTEDT1L  LFPTIVSMARTIEVISSGMLGSHSGTLQLMYKELQLLSPLVPTREFYFLRYCHQIEQGHW
FvEDT1    LFPTIVSMARTIEVISSGMLGSHSGTLQLMYKELQLLSPLVPTREFYFLRYCHQIEQGHW
          ************************************************************

FaTEDT1L  AIVDVSYDFPRDNQFANQSRSHRLPSGCLIQDMRNGYSKVTWVEHVEIEEKAPIHRLYRD
FvEDT1    AIVDVSYDFPRDNQFANQSRSHRLPSGCLIQDMPNGYSKVTWVEHVEIEEKAPIHRLYRD
          ********************************* **************************

FaTEDT1L  LIHSGQAFGAERWLVALQRMCERYACLMVSGTSTRDLEGVIPPPEGKRSMMKLAQRMVNN
FvEDT1    LIHSGQAFGAERWLVALQRMCERYACLMVSGTSTRDLEGVIPSPEGKRSMMKLAQRMVNN
          ****************************************** *****************

FaTEDT1L  FCASISTSNGHRWTTISGMNEVGVRVTIHKSTDPGQPNGVVLSAATTIWLPLSPQTVFNF
FvEDT1    FCASISTSNGHRWTTISGMNEVGVRVTIHKSTDPGQPNGVVLSAATTIWLPLSPQTVFNF
          ************************************************************

FaTEDT1L  FKDERTRPQWDVLSNGNAVQEVAHIANGSHPGNCISVLRAFNTSQNNMLILQESCIDSSG
FvEDT1    FKDERTRPQWDVLSNGNAVQEVAHIANGSHPGNCISVLRAFNTSQNNMLILQESCIDSSG
          ************************************************************

FaTEDT1L  SLVVYCPVDLPAINIAMSGEDPSYIPLLPSGFTITPDGRQDQGDTSASTSSCSNMNHIGG
FvEDT1    SLVVYCPVDLPAINIAMSGEDPSYIPLLPSGFTITPDGRQDQGDTSASTSSCSNINHIGG
          ******************************************************:*****

FaTEDT1L  SGSLITVAFQILVSSLPSAKLNMESVNTVNNLIGTTVQQIKSALNCSSS 709
FvEDT1    SGSLITVAFQILVSSLPSAKLNMESVNTVNNLIGTTVQQIKSALNCSSS 709
          *************************************************
```

Figure S3. Amino acid sequences comparison between the FaTEDT1L from octoploid strawberry (*F. × ananassa* Duchessne cv. *Toyonoka*) and the FvEDT1 from diploid strawberry (*F. vesca*). Amino acids at positions with "*" show complete matches between these two sequences.

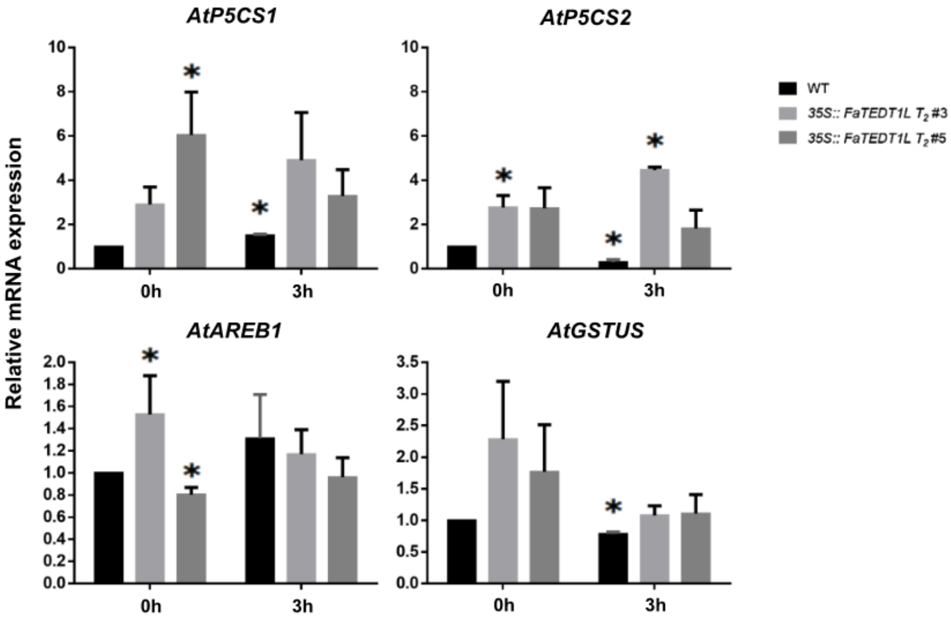

Figure S4. Gene expression analysis at the transcriptional level of *AtP5CS1*, *AtP5CS2, AtAREB1,* and *AtGSTUS* genes related to osmotic stress response was conducted through Real-time qPCR in *35S::FaTEDT1L* T_2 transgenic plants (#3 and #5) and wild-type (WT), respectively. The values detected in the untreated WT plants were set as 1, and the values determined in the untreated transgenic plants and other stress-treated plants were converted into relative ratios (n=3, *p<0.05).

A

AtSOS1 promoter region

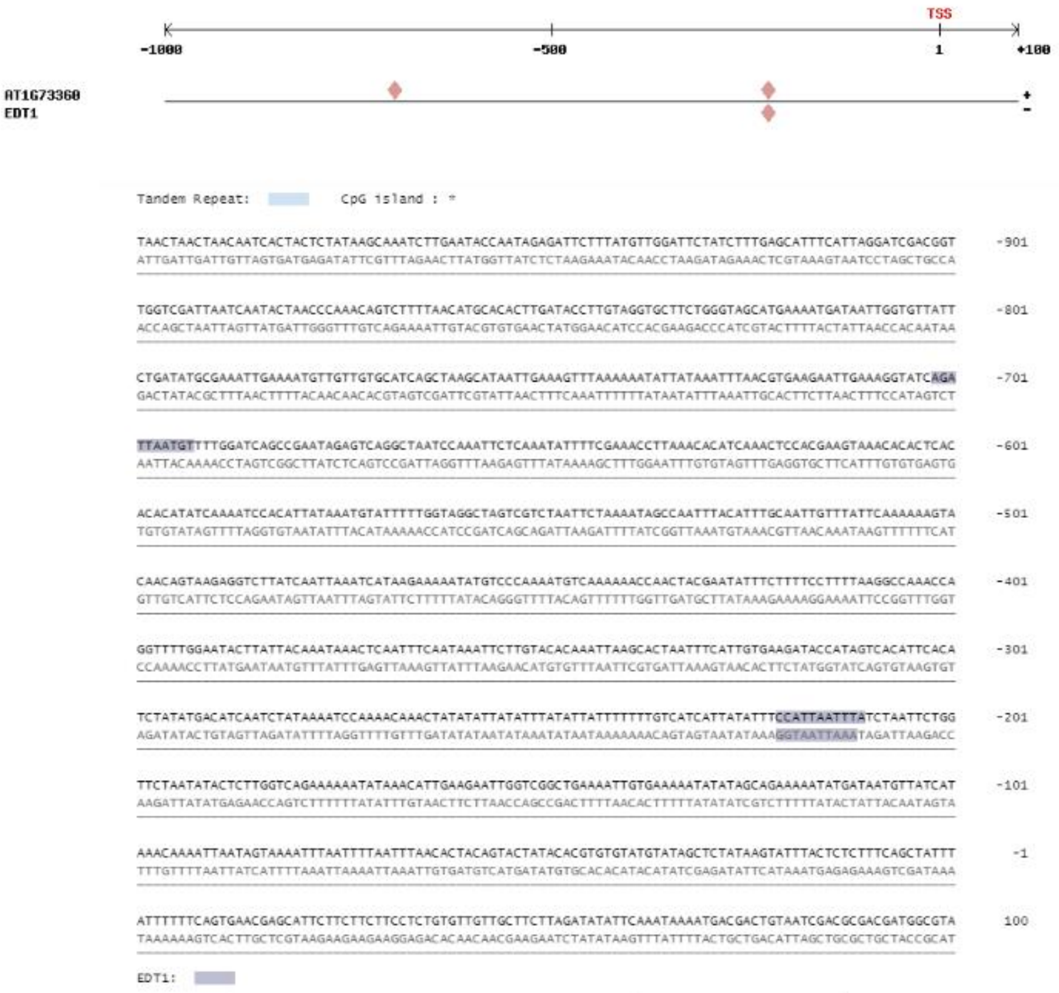

You can search Transcription Factor Binding Sites by using keywords: EDT1

Transcription Factor Binding Site

Homeodomain; HD-ZIP EDT1

Locus Name	Position	Strand	Sequence	Silmilar Score
AT1G73360	-703	+	agaTTAATgt	0.95
AT1G73360	-222	-	ccATTAAttt	0.97
AT1G73360	-222	+	ccATTAAttta	0.95

B

AtSOS2 promoter region

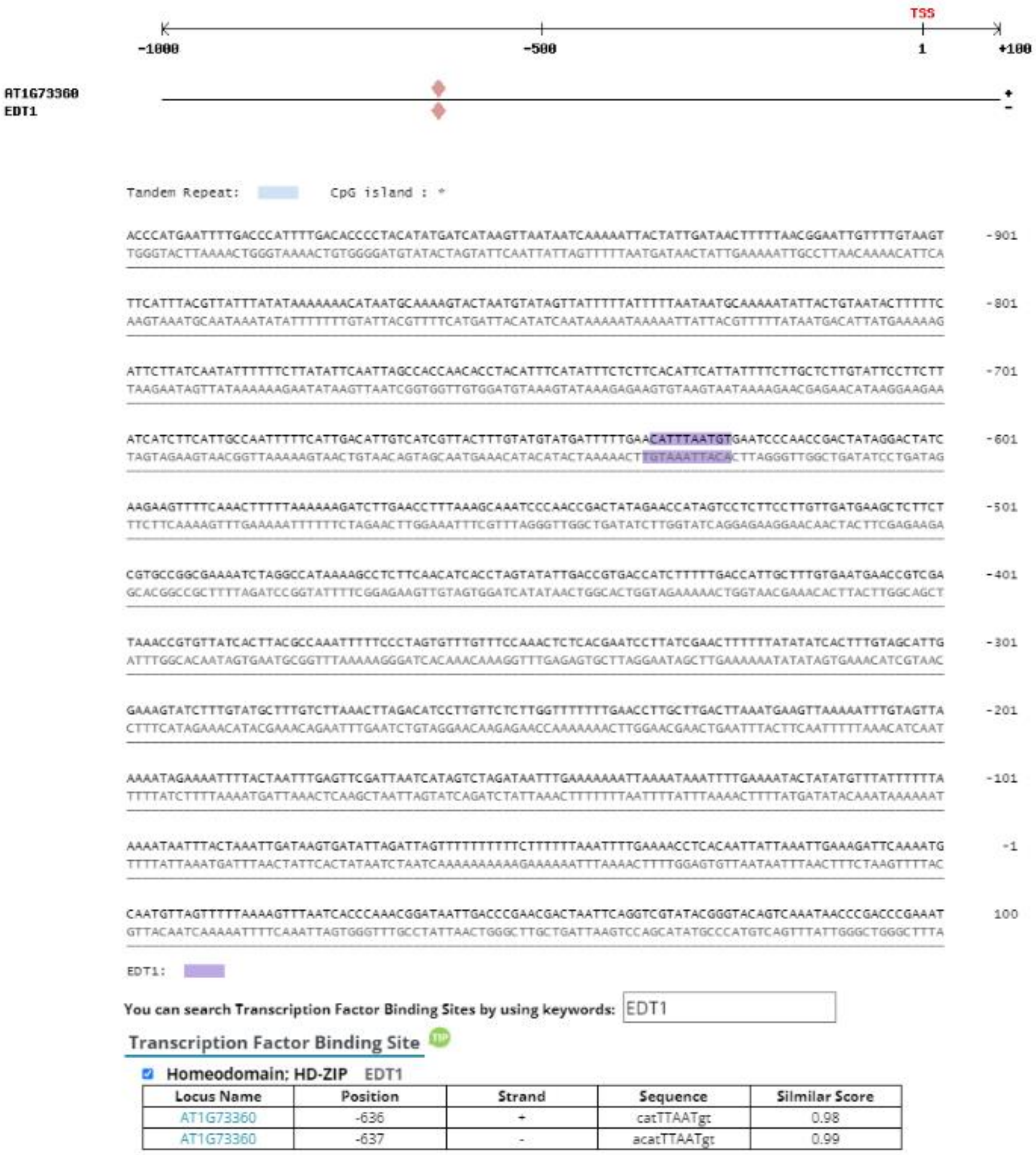

C

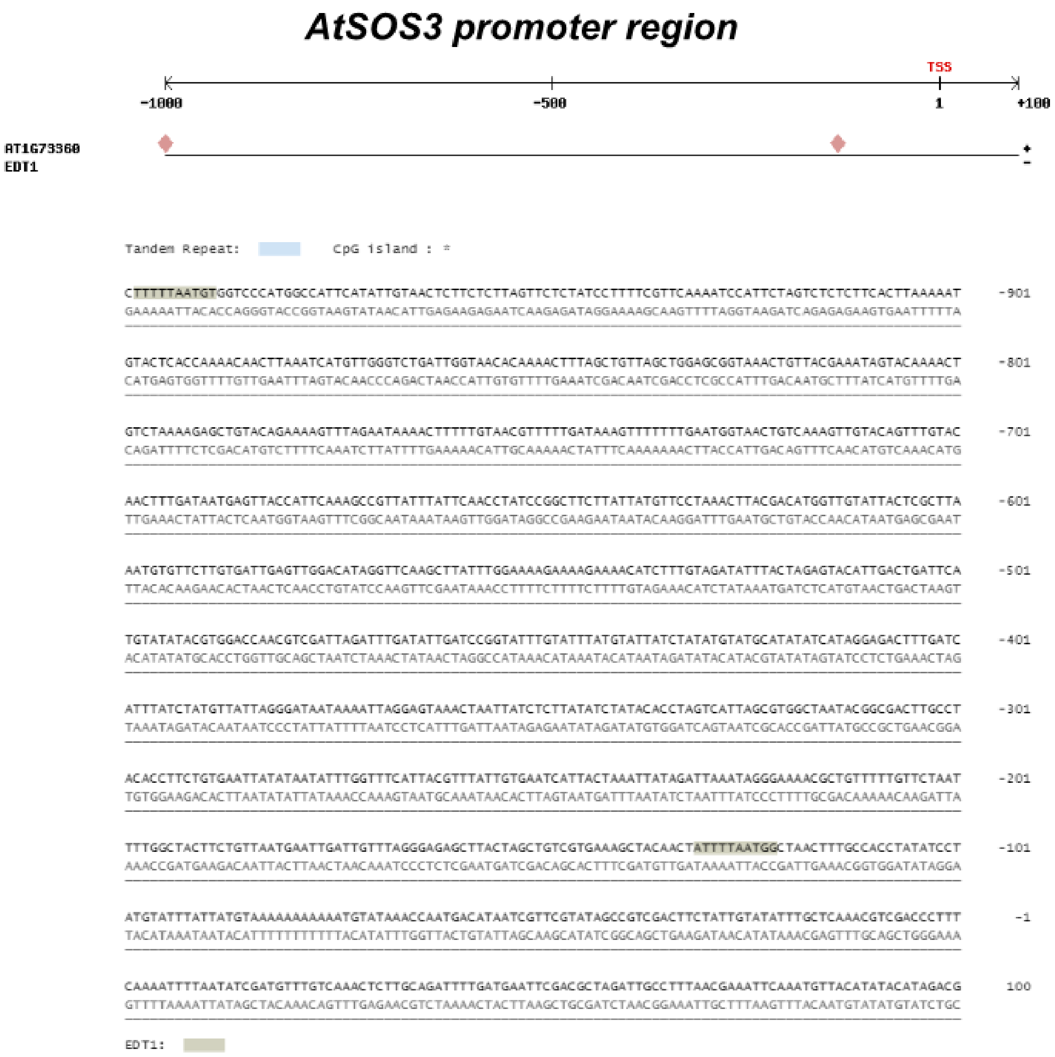

You can search Transcription Factor Binding Sites by using keywords: EDT1

Transcription Factor Binding Site

Homeodomain: HD-ZIP EDT1 (Conserved Region)

Locus Name	Position	Strand	Sequence	Silmilar Score
AT1G73360	-999	+	tttTTAATgt	0.94
AT1G73360	-132	+	attTTAATgg	0.93

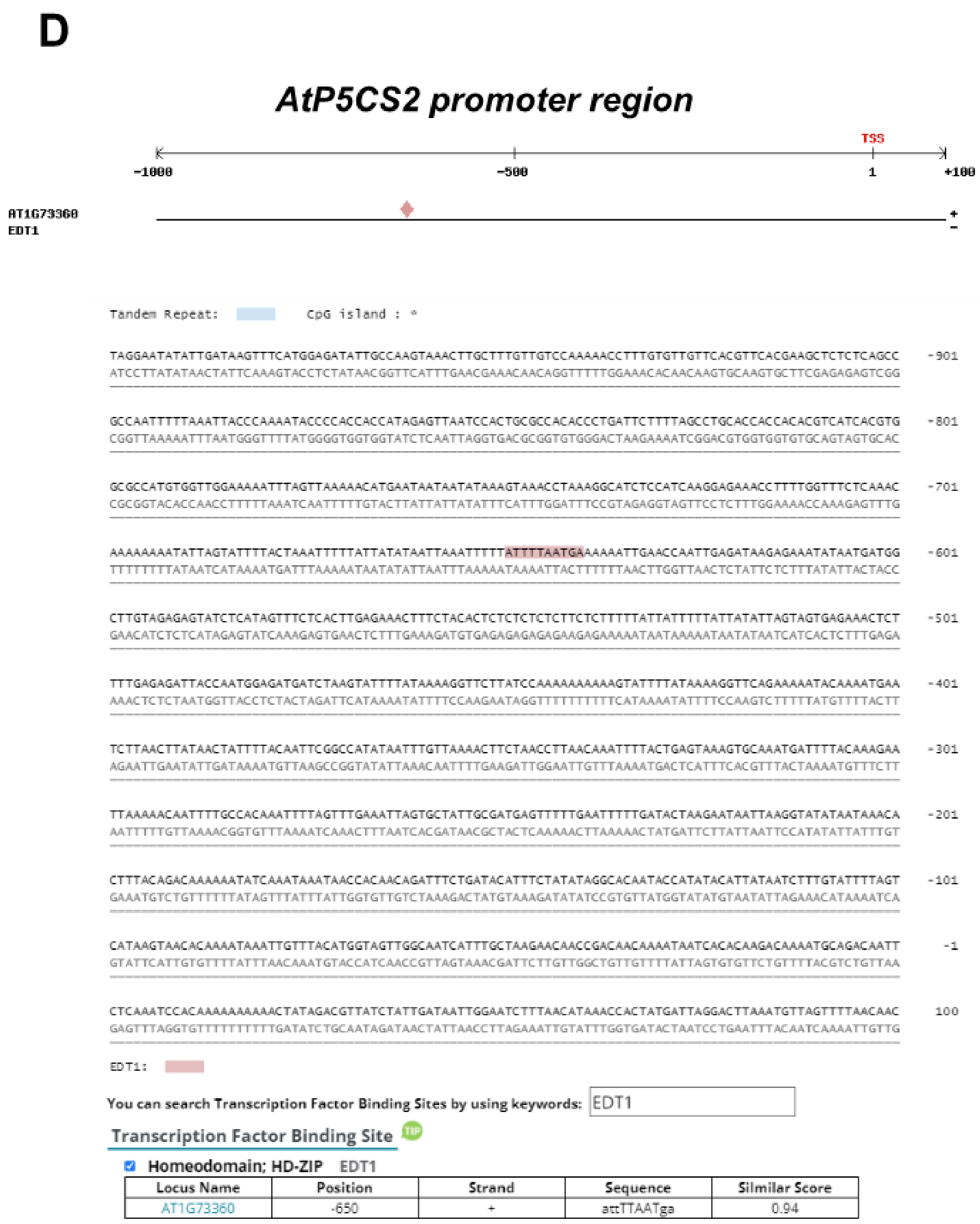

Figure S5: Prediction of the potential cis-acting elements for AtEDT1 transcription factor on *AtSOS1* (**A**), *AtSOS2* (**B**), *AtSOS3* (**C**), and *AtP5CS2* (**D**) by using the PlantPAN 4.0 bioinformatics platform.
